# Identification and Characterization of Perampanel Degradation Products: Stability-Indicating HPLC and In Silico Toxicological Assessment

**DOI:** 10.1021/acsomega.5c13204

**Published:** 2026-03-14

**Authors:** Jéssica Domingos da Silva, Gil Mendes Viana, Luana Gonçalves de Souza, Bárbara Abrahim-Vieira, Alessandra Mendonça Teles de Souza, Carina de Souza Anselmo, Henrique Marcelo Gualberto Pereira, Lucio Mendes Cabral, Valéria Pereira de Sousa

**Affiliations:** † Faculdade de Farmácia, Laboratório de Controle de Qualidade, 28125 Universidade Federal do Rio de Janeiro, Rio de Janeiro 21941-902, Rio de Janeiro, Brazil; ‡ Faculdade de Farmácia, Laboratório de Tecnologia Industrial Farmacêutica, Universidade Federal do Rio de Janeiro, Rio de Janeiro 21941-902, Rio de Janeiro, Brazil; § Faculdade de Farmácia, Laboratório de Modelagem Molecular & QSAR (ModMolQSAR), Universidade Federal do Rio de Janeiro, Rio de Janeiro 21941-902, Rio de Janeiro, Brazil; ∥ Instituto de Química, Laboratório Brasileiro de Controle de Dopagem LBCD–LADETEC, Universidade Federal do Rio de Janeiro, Rio de Janeiro 21941-902, Rio de Janeiro, Brazil

## Abstract

A stability-indicating HPLC-UV method enabling the simultaneous separation and monitoring of perampanel (PER) and all degradation products was developed and applied, providing adequate resolution and peak purity. Forced degradation studies were conducted to define the degradation profile of PER under acidic, alkaline, oxidative, photolytic, and metal-ion stress conditions. Five degradation products (DP-1 to DP-5) were identified by HPLC–UV and structurally characterized by HRMS/MS, and 1*D*/2D NMR spectroscopy (^1^H, ^13^C, COSY, and HSQC). Acidic hydrolysis produced a benzamide derivative (DP-1) and a benzoic acid derivative (DP-2), oxidative stress generated a pyridine *N*-oxide derivative (DP-3), and alkaline conditions yielded two previously unreported products, a ring-cleavage derivative of perampanel (DP-4) and a hydroxylated pyridone derivative of perampanel (DP-5). DP-1 was also detected during accelerated stability studies of PER tablets formulated with acidic excipients, demonstrating excipient–drug incompatibility under stressed storage conditions (40 °C/75% RH). The toxicity risk of the five degradation products was assessed using complementary in silico approaches (statistical and expert-rule methods) in accordance with ICH M7 guidance. None of the degradation products showed mutagenic concern and were therefore classified as ICH M7 Class 4 or Class 5 impurities. These results expand the structural knowledge of PER degradation products, clarify its main degradation pathways, and provide experimental and toxicological support for formulation development and impurity control.

## Graphical abstract

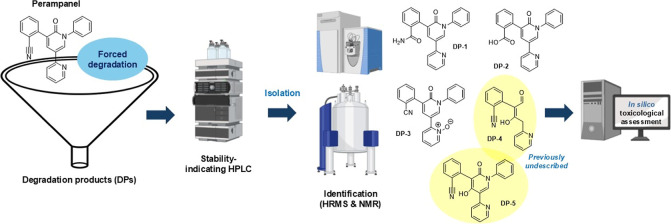

## 1 Introduction

Perampanel (PER), 2-(2-oxo-1-phenyl-5-(pyridin-2-yl)-1,2-dihydropyridin-3-yl) benzonitrile (C_23_H_15_N_3_O, molecular weight 349.38 g/mol), is a first-in-class, third-generation antiepileptic drug (AED) that acts as a selective, noncompetitive negative allosteric modulator of AMPA receptors.^1^ By inhibiting AMPA-mediated excitatory neurotransmission, PER decreases postsynaptic neuronal excitability and elevates the seizure threshold.^2^ Approved by the Food and Drug Administration (FDA) and European Medicines Agency (EMA) in 2012,^3^ PER is indicated for primary generalized tonic-clonic seizures and focal-onset seizures, with or without secondary generalization, in patients ≥12 years of age.

International Council for Harmonisation (ICH) guidelines, including Q1A, Q3A, and Q3B, emphasize the importance of impurity profiling and forced degradation studies during drug development. These guidelines establish requirements for the identification, qualification, and control of impurities, including both process-related impurities and degradation products (DPs) formed under stress conditions.^4,5^ Forced degradation studies are essential to evaluate the intrinsic stability of drug substances, to elucidate degradation pathways, and to support the development of stability-indicating analytical methods. Comprehensive characterization of DPs is critical for toxicological assessment, impurity qualification, and quality control throughout the drug life cycle. According to ICH Q3A and Q3B, impurities above-defined identification and qualification thresholds must be structurally characterized and assessed for safety, highlighting the need for reliable analytical approaches. This is particularly relevant for recently introduced drugs such as PER, for which official pharmacopoeial monographs and comprehensive degradation profiles are still lacking. In this context, forced degradation studies play a key role in impurity profiling by generating relevant DPs under controlled stress conditions, thereby supporting both regulatory compliance and the development of robust analytical methods.^6,7^

Although some studies have explored the forced degradation of PER,^8–12^ its degradation pathways remain incompletely elucidated. Previous reports have shown that PER is susceptible to acidic and alkaline hydrolysis, oxidation, photolysis, and thermal stress; however, the number and identity of DPs reported vary significantly among studies. For instance, Saida et al. (2018)^9^ identified only two DPs under acidic and oxidative conditions and did not observe degradation under alkaline, photolytic, or thermal stress. In contrast, Xia et al. (2020)^11^ reported five DPs formed under acidic, alkaline, and oxidative conditions; however, only two were fully characterized. Importantly, none of the available studies have evaluated the toxicological relevance of PER degradation products, leaving a critical gap in the impurity safety assessment required by regulatory guidelines. These methodological differences and limitations underscore the need for a comprehensive study integrating systematic forced degradation, advanced structural characterization, and toxicological evaluation of degradation products, as proposed in the present work.

In silico toxicological assessment has become an integral component of impurity evaluation and is explicitly recommended in ICH M7 for the assessment of DNA-reactive impurities. The guideline endorses the combined use of complementary statistical models and expert-rule systems, provided they comply with OECD (Organisation for Economic Co-operation and Development) validation principles. Because degradation impurities are often formed in low amounts and are not always amenable to experimental Ames testing, computational approaches represent a practical and scientifically justified alternative for preliminary mutagenicity assessment.^13,14^ In recent years, QSAR-based in silico toxicology approaches have been widely applied in pharmaceutical development and impurity risk assessment to support early safety decision-making, reduce experimental costs, and minimize animal testing, in line with current regulatory and ethical expectations. To date, no systematic in silico toxicological evaluation of PER degradation products has been reported.

The aim of this study was to establish a comprehensive degradation profile of PER through forced degradation studies under acidic, alkaline, oxidative, photolytic, and metal-ion stress conditions in accordance with ICH recommendations.^4,5^ The specific objectives included developing and applying a stability-indicating HPLC–UV method for monitoring PER and its degradation products; evaluating excipient–drug compatibility through accelerated stability testing of PER tablets formulated with excipients of acidic, alkaline, or oxidative potential; and assessing the toxicological profile of each degradation product using complementary in silico methodologies, including mutagenicity evaluation in accordance with ICH M7 guidelines.^15,16^ Collectively, these objectives support the elucidation of PER degradation pathways and provide a regulatory framework for impurity safety assessment and formulation development.

## 2 Materials and Methods

### 2.1 Chemical and Reagents

Perampanel hydrate (purity >99.9%) was obtained from ShangHai Biochempartner Co. HPLC-grade acetonitrile, methanol, and glacial acetic acid were purchased from Loba Chemie and TEDIA. Analytical-grade sodium hydroxide, hydrochloric acid, hydrogen peroxide, urea-hydrogen peroxide, sodium carbonate, tartaric acid, and cupric sulfate were obtained from Sigma-Aldrich. Magnesium stearate and microcrystalline cellulose were supplied by Farmos Industry. Deuterated dimethyl sulfoxide (DMSO-*d*_6_, 99.9% + 0.05% TMS) was acquired from Cambridge Isotope Laboratories. Sample filtration was performed using 0.45 μm PTFE filters (Sorbline). Purified water was produced using a Milli-Q system.

### 2.2 Forced Degradation Studies

Forced degradation studies were conducted by exposing approximately 5 mg of PER to acidic, alkaline, oxidative, metal-ion, and photolytic stress conditions in accordance with ICH Q1B and Q3A­(R2) guidelines. Samples obtained from acidic and alkaline hydrolysis were neutralized prior to HPLC analysis. All stressed samples were subsequently diluted with ACN/H_2_O (60:40 v/v) to ensure compatibility with the chromatographic mobile phase and filtered through 0.45 μm PTFE membranes before HPLC-DAD and HPLC-HRMS analysis.

#### 2.2.1 Acidic Hydrolysis

Acidic hydrolysis was performed by exposing PER to 0.5 M HCl under reflux for 14 h. Due to the good solubility of PER in acidic media, this stress condition was carried out directly in 10 mL of aqueous stress solution, without the addition of organic solvent.

#### 2.2.2 Alkaline Hydrolysis

Alkaline hydrolysis was conducted using 1 M NaOH under reflux for 24 h. As PER is insoluble in water but readily soluble in organic solvents, alkaline stress was performed using a mixture of the aqueous stress solution and methanol (20:80 v/v, 10 mL) to ensure complete dissolution of the drug substance.

#### 2.2.3 Oxidative Degradation

Oxidative degradation was induced by treating PER with 15% hydrogen peroxide (H_2_O_2_) under reflux for 24 h. In this condition, a mixture of the oxidative stress solution and methanol (20:80 v/v, 10 mL) was employed to maintain adequate solubility of PER throughout the experiment.

#### 2.2.4 Metal-Ion Degradation

Metal-ion-induced degradation was evaluated by exposing PER to 25 mM CuSO_4_ 5H_2_O under reflux for 72 h. Similar to other stress conditions, a mixture of the metal-ion stress solution and methanol (20:80 v/v, 10 mL) was used to ensure proper solubilization of the drug.

#### 2.2.5 Photolytic Degradation

Photodegradation studies were performed in a photostability chamber (model 424-CF, Ethiktechnology) following ICH Q1B Option 2. Solid PER (200 mg) and PER solutions in MeOH/ACN (1:1 v/v, 10 mg/mL) were placed in inert glass containers and exposed to a total illumination of 1.2 × 10^6^ lux/h of fluorescent light and 200 W h m^–2^ of near-UV radiation. Control samples (solid and solution) were protected from light but kept under identical environmental conditions.

### 2.3 Accelerated Stability Studies

Prototype tablets were prepared by direct compression and contained 20 mg of PER, 100 mg of microcrystalline cellulose, and 0.1% magnesium stearate. To assess excipient–drug compatibility under stress conditions, tablets were additionally formulated with 10 mg of tartaric acid (acidic medium), sodium carbonate (alkaline medium), or urea–hydrogen peroxide (oxidative medium). Test and control tablets were stored either in amber glass bottles sealed with Parafilm (low-permeability) or wrapped in kraft paper (semipermeable)^12^ and placed in a stability chamber at 40 °C and 75% RH for up to 24 months. Samples were withdrawn at 0, 3, 6, 12, and 24 months, dissolved in methanol, diluted with ACN/H_2_O (60:40 v/v), and analyzed by HPLC-DAD and HPLC-HRMS.

### 2.4 Chromatography

Chromatographic analyses were performed on an Elite LaChrom HPLC system (Merck-Hitachi) equipped with a quaternary pump (L-2130), autosampler (L-2200), column oven (L-2300), and diode-array detector (L-2455). Separation was achieved on an XTerra C18 MS column (25.0 × 4.6 mm, 5 μm; Waters) maintained at 25 °C and acidic buffer. Mobile-phase composition and gradient conditions were adapted from Franco et al. (2016).^17^ The mobile phase consisted of acetonitrile (A) and water acidified to pH 3.0 with acetic acid (B). The gradient program was as follows: 0–10 min, 30–40% A; 10–15 min, 40–60% A; 20–23 min, 60–30% A; 25 min, 30% A. The flow rate was 1.0 mL min^–1^, the injection volume was 20 μL, and detection was performed at 290 nm. Chromatographic data acquisition and system control were performed using EZChrom Elite software (Agilent Technologies). These chromatographic conditions provided baseline separation of PER and all degradation products and were also used for preparative HPLC and HPLC-HRMS analyses.

### 2.5 HRMS and Fragmentation Analysis of PER and DPs

HRMS analyses were performed using a Thermo Scientific Dionex Ultimate 3000 HPLC system coupled to a Q Exactive Hybrid Quadrupole-Orbitrap mass spectrometer equipped with an electrospray ionization (ESI) source. The instrument was calibrated daily using the standard calibration solution supplied by the manufacturer.

The ESI source parameters were as follows: capillary temperature, 300 °C; spray voltage, 3.5 kV; sheath gas, 65; auxiliary gas, 20; and sweep gas, 4 arbitrary units. Full-scan MS spectra were acquired in positive ion mode over *m*/*z* 50–750 at a resolution of 70,000 fwhm, with an AGC target of 3 × 10^6^ and a maximum injection time of 200 ms. Data were processed using TraceFinder 3.2 software (Thermo Scientific).

Tandem MS analyses (Full MS/MS) were carried out in positive ion mode using data-independent acquisition (DIA). An inclusion list containing the exact masses of PER and all DPs was generated from full-scan MS data. Product-ion spectra were recorded using a normalized collision energy (NCE) of 35 eV. DIA spectra were acquired at a resolution of 35,000 fwhm (*m*/*z* 200), with an AGC target of 2 × 10^5^, an isolation window of 3.0 *m*/*z*, and an NCE step of 30 ± 10 eV. Spectral interpretation was performed using Xcalibur Qual Browser 3.0.63.

### 2.6 Isolation of Degradation Products

DP-2 was isolated after subjecting PER to complete degradation in 6 M HCl under reflux for 1 h in order to increase its formation and facilitate isolation. DP-1, DP-3, DP-4, and DP-5 were isolated by preparative HPLC because PER remained present in the partially degraded samples obtained under stress conditions. Preparative separations were performed using the analytical gradient method (Section 2.4) scaled to a flow rate of 21 mL/min on a Luna Prep C18 column (250.0 × 21.2 mm, 10 μm, 100 Å; Phenomenex) with an injection volume of 2.5 mL. Collected fractions were concentrated by rotary evaporation to remove mobile-phase solvents.

### 2.7 Nuclear Magnetic Resonance Spectroscopy—NMR

NMR analyses of PER and all isolated DPs were performed to confirm the structures proposed by HRMS. One-dimensional ^1^H and ^13^C NMR spectra were recorded on a 400 MHz Varian instrument, and chemical shifts (δ) and coupling constants (*J*) are reported in ppm and Hz, respectively. Two-dimensional COSY, TOCSY, and HSQC experiments were acquired on a 500 MHz Varian VNMRSYS-500 instrument. All spectra were recorded in DMSO-*d*_6_ at ambient temperature.

### 2.8 In Silico Toxicity Risk Assessment

The 2D chemical structures of each DP were evaluated for mutagenicity and carcinogenicity in accordance with ICH M7 requirements. Predictions were generated using ADMET Predictor (v9.5; statistical model, Method I) and ACD/Laboratories Percepta (v2021; expert-rule system, Method II). To increase the robustness of the assessment, a third evaluation was performed using the VEGA HUB platform, which incorporates models developed in EU projects such as CAESAR and the U.S. EPA TEST tool. Additional end points, including hepatotoxicity, hERG blockage, and acute oral toxicity (LD_50_), were also predicted as described previously.^18–21^

## 3 Results and Discussion

### 3.1 Optimization of Chromatographic Conditions

Method development was performed in parallel with the degradation studies to obtain chromatographic conditions suitable for monitoring PER and all resulting DPs. The development of a stability-indicating HPLC–UV method focused on achieving adequate separation between perampanel (PER) and its degradation products generated under different stress conditions. A volatile mobile phase consisting of acetonitrile and water acidified to pH 3.0 using acetic acid (analytical grade) was selected to ensure compatibility with both DAD detection and ESI-HRMS.^22,23^ UV detection at 290 nm provided adequate sensitivity for PER without interfering contributions from stress reagents, diluents, or mobile-phase components.

Acetonitrile was selected as the organic component because its low UV cutoff (190 nm) avoids interference at the detection wavelength of 290 nm.^17^ Initial isocratic conditions (60% acetonitrile, 1.0 mL/min) did not provide adequate separation of PER and its DPs, resulting in significant peak overlap due to the wide polarity range of the degradation products. Therefore, the chromatographic conditions were systematically optimized by adjusting the elution mode and gradient profile to improve resolution and peak shape. Gradient elution (30–60% acetonitrile over 15 min) provided baseline resolution of all degradation products, with chromatographic resolution values >2.0 and DAD peak purity indices >0.90.

The optimized conditions described in Section 2.4 were subsequently applied to preparative HPLC and HRMS analyses with consistent performance. Representative chromatograms and the retention times of DP-1 to DP-5 are shown in Figure 1.

**Figure 1:**
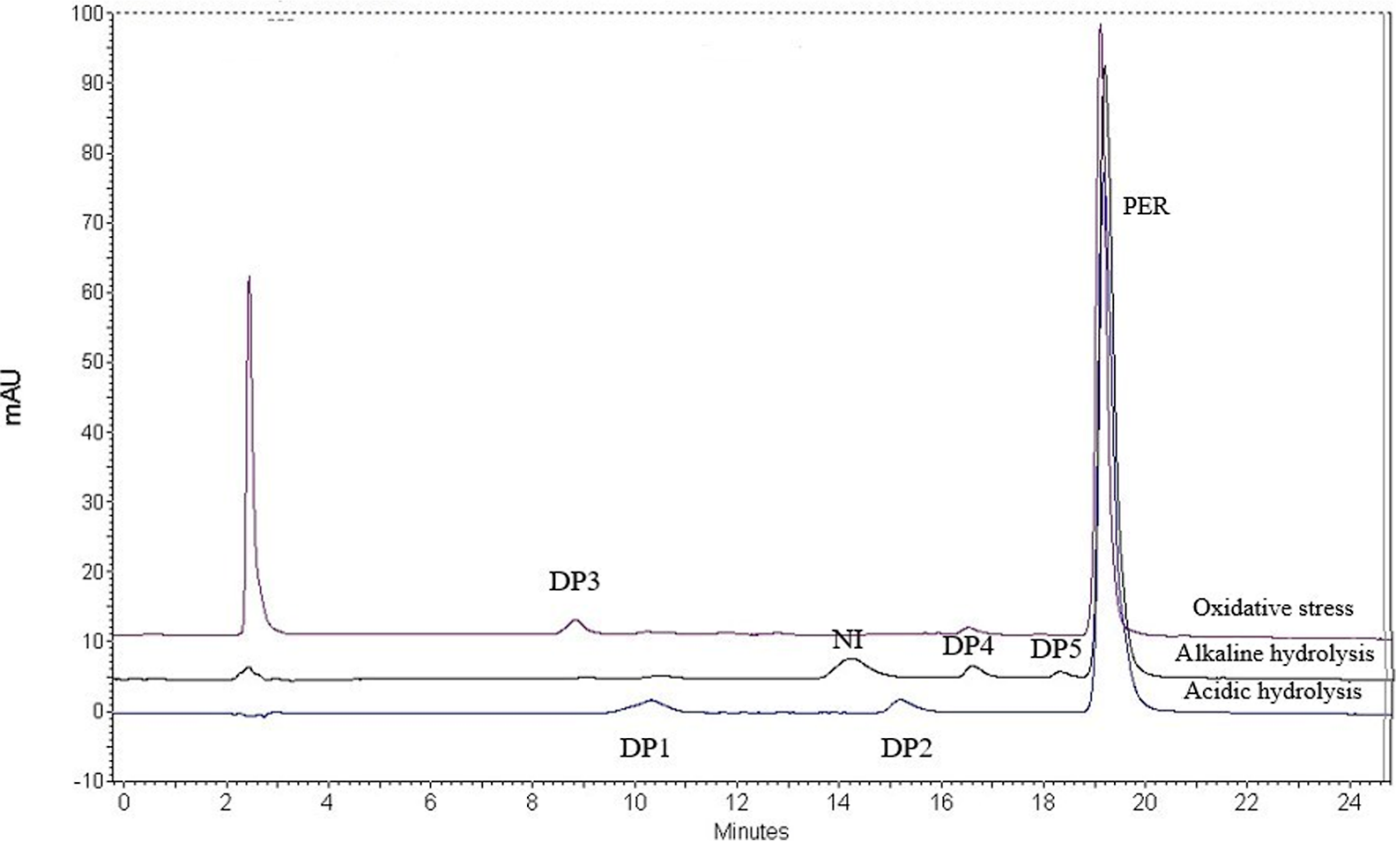
Chromatograms obtained with optimal analysis conditions using the UV-DAD detector for acid, alkaline, and oxidative stress; C18 column, 4.6 × 25 cm, mobile phase: acetonitrile (mobile phase A) and acidified water pH 3.0 with acetic acid (mobile phase B) with the following gradient T­(min)/% A, 0–10/30–40, 10–15/40–60, 20–23/60–30, 25/30. Flow: 1 mL/min and detection at 290 nm. NI—Not identified.

The optimized analytical HPLC conditions were directly transferred to preparative HPLC to isolate individual degradation products for NMR analysis. This approach ensured chromatographic selectivity consistent with the analytical method while enabling efficient fraction collection. Because complete degradation of PER was not achieved under most stress conditions, preparative separations were designed to resolve degradation products from residual parent compound. Despite these challenges, sufficient amounts of all degradation products were successfully isolated for structural characterization.

High-resolution mass spectrometry (HRMS) conditions were optimized to support accurate mass determination and structural elucidation of PER and its degradation products. An Orbitrap-based analyzer was selected due to its high mass accuracy and resolving power, enabling reliable elemental composition assignment for both precursor and fragment ions. Data-independent acquisition combined with targeted inclusion lists was employed to ensure consistent MS/MS fragmentation of all detected degradation products, including low-abundance species.

The HRMS/MS data were interpreted based on accurate mass measurements and characteristic fragmentation patterns, providing key structural information for the proposed degradation products. This approach, combined with chromatographic behavior and NMR data, enabled confident structural elucidation of PER degradation products.

### 3.2 Degradation Behavior of Perampanel Drug Substance

PER was subjected to acidic, alkaline, oxidative, photolytic, and metal-ion stress conditions in accordance with ICH guidelines.^3,18–20^ Because PER exhibits good solubility in acidic aqueous media and organic solvents such as methanol, acid hydrolysis was performed in aqueous solution, whereas all other stress conditions used a mixture of stress reagent and methanol (20:80 v/v). The experimental conditions that resulted in 10–30% degradation, as recommended for forced degradation studies, and the corresponding DPs formed under each condition are summarized in Table 1.

**Table 1:** Forced Degradation Conditions of Perampanel and Corresponding Degradation Products Identified under Each Stress Condition

stress condition	degradation agent	experimental conditions	time (h)	degradation products (relative peak area, %)^a^
acid hydrolysis	0.5 M HCl	reflux	14	DP-1 (13.65) and DP-2 (6.19)
ixidation	15% H_2_O_2_ in MeOH (50:50)	reflux	24	DP-3 (12.92)
alkaline hydrolysis	1 M NaOH in MeOH (20:80)	reflux	24	DP-4 (2.47) and DP-5 (0.83)
metal ion	25 mM CuSO_4_	reflux	48	no degradation products observed
photolytic stress	fluorescent light and near-UV radiation	1.2 × 10^6^ lux/h and 200 W h m^–2^	72	no degradation products observed

a Relative peak area (%) calculated by area normalization within each stress condition (sum of PER and DPs = 100%), assuming similar UV response at 290 nm.

No degradation products were detected under photolytic stress, indicating that PER is photostable under ICH Q1B conditions. Likewise, exposure to metal ions (Cu^2+^) for 48 h under reflux resulted in no detectable degradation, with PER recovery close to 100%.

### 3.3 Characterization of the Drug

The characteristic fragmentation spectrum of PER is shown in Figure S1 (Supporting Information), and the corresponding accurate masses and elemental compositions are summarized in Table S1. Consistent with previous reports,^24–28^ the MS/MS spectrum of PER (Figure S1) showed the protonated molecular ion at *m*/*z* 350.12827 (C_23_H_16_N_3_O^+^). A diagnostic product ion at *m*/*z* 104.04972 (C_7_H_6_N^+^) is consistent with a benzonitrile-derived fragment (Figure S31). The complementary fragment at *m*/*z* 247.08630 (C_16_H_11_N_2_O^+^) supports cleavage between the benzonitrile ring and the remaining PER scaffold. Furthermore, the ion at *m*/*z* 219.09134 (C_15_H_11_N_2_^+^) can be rationalized by CO loss (−28 Da) from the pyridone-containing fragment, corroborating the proposed fragmentation pathway.

The ^1^H NMR spectrum of PER (Figure S2, Table S2) showed a characteristic pattern of aromatic and heteroaromatic protons, allowing the identification of the benzonitrile, pyridine, pyridone, and phenyl moieties. The pyridine ring was identified by two doublets at δ 8.59 (H-24) and δ 8.01 ppm (H-21), a triplet of doublets at δ 7.84 ppm (H-22), and another at δ 7.30 ppm (H-23). The pyridone ring protons were observed as two doublets at δ 8.53 (H-10) and δ 8.48 ppm (H-8), consistent with the conjugated heterocyclic system.

The benzonitrile ring was assigned based on three doublets at δ 7.93 (H-2), 7.79 (H-6), and 7.72 ppm (H-5), together with a multiplet at δ 7.63–7.55 ppm (H-1). The phenyl ring protons appeared as a multiplet in the same region (δ 7.63–7.55 ppm for H-14, H-18, H-15, and H-17), with H-16 appearing as a multiplet at δ 7.52 ppm. The proton connectivities within each aromatic system were confirmed by the COSY spectrum (Figure S4).

The ^13^C NMR spectrum of PER (Figure S3, Table S2) further supported the proposed structure. The carbonyl carbon of the pyridone ring (C-12) was observed at δ 159.96 ppm. The nitrile carbon (C-26) appeared at δ 118.73 ppm, which is diagnostic of a −C≡N group. The aromatic carbons of the benzonitrile, pyridone, pyridine, and phenyl rings were distributed between δ 117 and 153 ppm, in agreement with the highly conjugated system.

The HSQC spectrum (Figure S5) allowed the unambiguous correlation of each proton with its directly attached carbon, confirming the assignments made from the ^1^H and ^13^C NMR spectra. Taken together, the HRMS data, the characteristic chemical shifts in the ^1^H and ^13^C NMR spectra, and the COSY and HSQC correlations provide consistent and unequivocal support for the proposed structure of PER.

### 3.4 Characterization of Degradation Products

High-resolution tandem mass spectrometry (HRMS/MS) has become a key analytical approach for the structural elucidation of pharmaceutical impurities and degradation products, particularly when combined with accurate-mass measurements and systematic interpretation of fragmentation pathways. Recent studies have demonstrated the application of LC–HRMS/MS for the identification of degradation products, often in combination with complementary spectroscopic techniques such as NMR, enabling confident structural assignments.^29–32^

In particular, the analysis of diagnostic product ions and characteristic neutral losses has been recognized as a critical strategy for differentiating closely related or isomeric compounds analyzed by LC–HRMS/MS.^33^ Furthermore, recent studies highlight the central role of high-resolution mass spectrometry in impurity profiling, allowing the identification of trace-level degradation products and supporting regulatory requirements in pharmaceutical development.^34,35^

For perampanel (PER), the available literature reports only a limited number of degradation products identified using LC-based techniques, with limited interpretation of fragmentation pathways.^3,9,11,26^ In the present study, HRMS/MS data were interpreted in combination with chromatographic behavior (HPLC–UV) and 1*D*/2D NMR spectroscopy, enabling reliable structural elucidation of PER and its degradation products.

#### 3.4.1 Acid Hydrolysis Degradation Products

DP-1 was formed under acidic hydrolysis and exhibited a protonated ion at *m*/*z* 368.13943 (C_23_H_18_N_3_O_2_^+^). The major product ion at *m*/*z* 351.11213 (C_23_H_15_N_2_O_2_^+^) corresponds to a neutral loss of 17 Da, consistent with NH_3_ loss from an amide group, supporting the conversion of the benzonitrile moiety into a benzamide (Table S1; Figures S6 and S32). Additional fragments at *m*/*z* 248.07013 (C_16_H_10_NO_2_^+^) and 220.07569 (C_15_H_10_NO^+^) are consistent with fragmentation within the remaining aromatic scaffold, including a CO loss (−28 Da) from the oxygenated fragment. Overall, the MS/MS data agree with the NMR evidence (appearance of a new carbonyl signal and disappearance of the nitrile carbon), confirming DP-1 as the benzamide derivative.

The ^1^H NMR spectrum of DP-1 (Figure S7, Table S3) showed the appearance of new signals at δ 7.65–7.39 ppm (multiplet) and δ 7.16 ppm (singlet), which were assigned to the amide NH protons. These signals were absent in the PER spectrum, confirming the formation of an amide group. Importantly, the remaining aromatic and heteroaromatic proton patterns were largely preserved, indicating that the core scaffold of PER remained intact.

In the ^13^C NMR spectrum of DP-1 (Figure S8, Table S3), the disappearance of the nitrile carbon signal (δ 118.73 ppm in PER) and the appearance of a new carbonyl carbon at δ 170.90 ppm (C-26) provided decisive evidence for the hydrolysis of the nitrile group into an amide. This chemical shift is characteristic of amide carbonyl carbons and is fully consistent with the proposed transformation.

The COSY spectrum (Figure S9) confirmed that the proton–proton correlations within the aromatic and heteroaromatic systems were preserved, indicating that no major rearrangements occurred in these moieties. The HSQC spectrum (Figure S10) further supported the assignments by correlating each proton with its directly attached carbon, allowing the unambiguous identification of the modified region and the preserved substructures.

Taken together, the HRMS data, the diagnostic MS/MS fragmentation, the appearance of new amide proton and carbonyl signals in the NMR spectra, and the preserved COSY and HSQC correlation patterns unequivocally support the identification of DP-1 (2-(2-oxo-1-phenyl-5-(pyridin-2-yl)-1,2-dihydropyridin-3-yl) benzamide; Figure 2), previously identified by Saida et al. (2018).^3,9^

**Figure 2:**
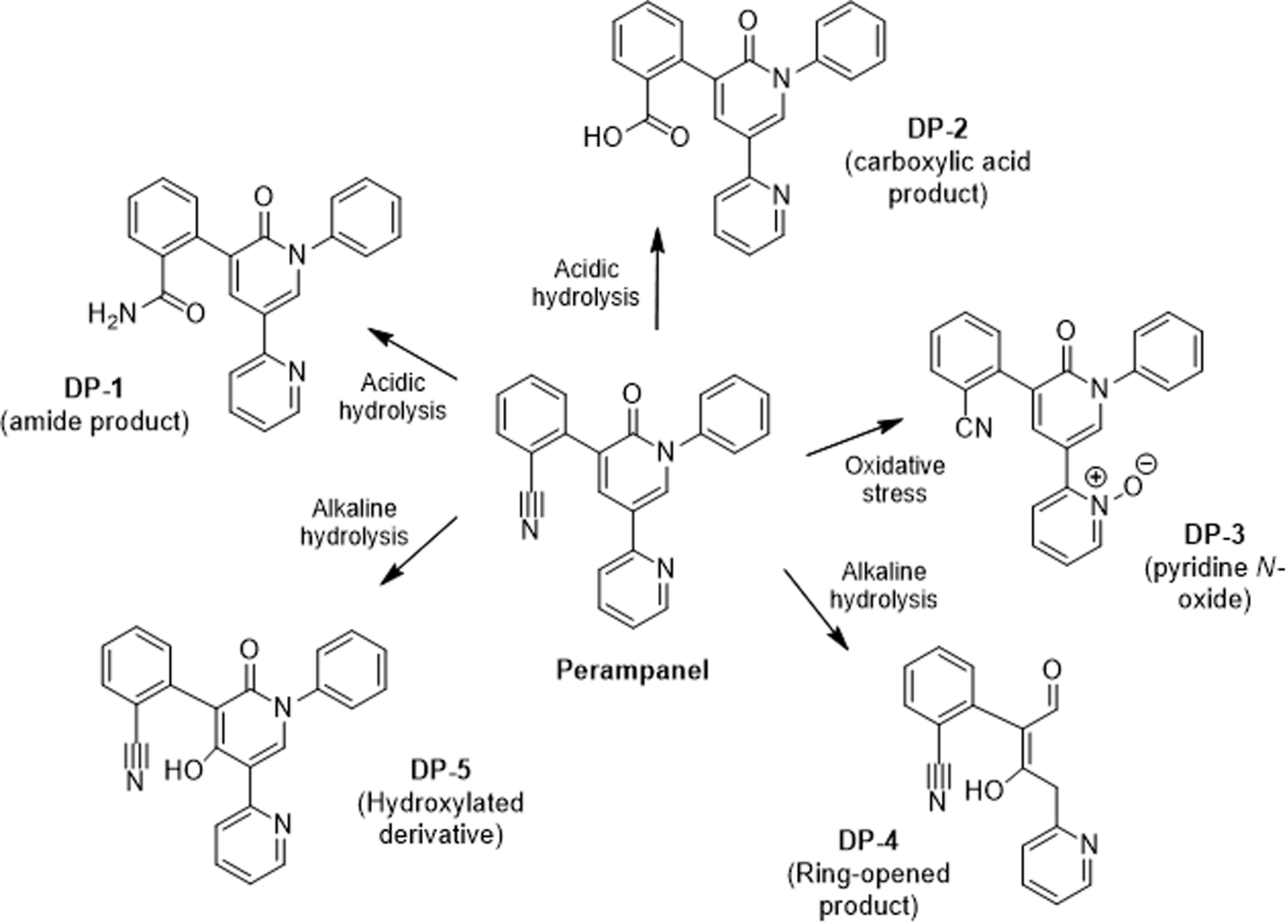
Proposed degradation products of Perampanel under forced degradation conditions. Acidic hydrolysis led to the formation of DP-1 and DP-2, oxidative stress generated DP-3, and alkaline hydrolysis resulted in the formation of DP-4 and DP-5.

DP-2 was also formed under acidic hydrolysis and showed a protonated molecular ion at *m*/*z* 369.12270 (C_23_H_17_N_2_O_3_^+^). The MS/MS spectrum displayed a fragment at *m*/*z* 351.11197 (C_23_H_15_N_2_O_2_^+^), corresponding to a neutral loss of 18 Da (H_2_O), supporting conversion of the nitrile into an oxygenated benzoic acid derivative. Further fragmentation yielded *m*/*z* 323.11687 (C_22_H_15_N_2_O^+^), consistent with additional loss of a small neutral CO. The oxygenated aromatic fragment pair *m*/*z* 248.06979 → 220.07497 (−28 Da) is consistent with CO loss from a carbonyl-containing ion (Table S1; Figures S11 and S33).

The ^1^H NMR spectrum of DP-2 (Figure S12, Table S4) showed a general preservation of the aromatic and heteroaromatic proton patterns associated with the pyridine, pyridone, and phenyl moieties, indicating that the main scaffold of PER was retained. However, the overlapping of signals in the aromatic region made direct assignment of all protons difficult. The COSY spectrum (Figure S14) was therefore crucial for establishing the proton–proton connectivities within each aromatic system, enabling the identification of the pyridine and pyridone ring protons.

The ^13^C NMR spectrum of DP-2 (Figure S13, Table S4) provided decisive evidence for the proposed structure. The disappearance of the nitrile carbon signal observed in PER (δ 118.73 ppm) and the appearance of a new carbonyl signal at δ 168.95 ppm (C-26) confirmed the transformation of the nitrile into a carboxylic acid group. This chemical shift is fully consistent with carboxylic acid carbonyl carbons. The remaining aromatic and heteroaromatic carbon signals were largely conserved, indicating that no major rearrangements occurred in these regions.

The HSQC spectrum (Figure S15) allowed the direct correlation of each proton with its attached carbon, which confirmed the assignments derived from the ^1^H and ^13^C NMR spectra and validated the preservation of the original aromatic framework. Taken together, the HRMS data, the characteristic MS/MS fragmentation behavior, and the diagnostic NMR changes unequivocally support the identification of DP-2 (2-(2-oxo-1-phenyl-5-(pyridin-2-yl)-1, 2-dihydropyridin-3-yl) benzoic acid; Figure 2), consistent with previously reported acidic hydrolysis of PER.^11,26^

The degradation behavior of PER under the applied stress conditions can be rationalized based on well-established chemical transformation pathways. Under acidic conditions, the degradation of PER proceeds via hydrolysis of the nitrile group. The mechanism involves nucleophilic attack of water on the electrophilic carbon of the nitrile, leading to the formation of an amide intermediate (DP-1). Further hydrolysis of this intermediate yields the corresponding carboxylic acid derivative (DP-2). Such sequential conversion from nitrile to amide and subsequently to carboxylic acid is a well-known acid-catalyzed transformation and is consistent with the structures elucidated by HRMS/MS and NMR analyses.

#### 3.4.2 Oxidative Degradation Product

DP-3 exhibited [M + H]^+^ at *m*/*z* 366.12324 (C_23_H_16_N_3_O_2_^+^), consistent with mono-oxygenation relative to PER (Table S1). The product ion at *m*/*z* 349.12041 (C_23_H_15_N_3_O^+^) corresponds to a neutral loss of 17 Da, a characteristic behavior reported for protonated aromatic *N*-oxides, supporting oxidation of the pyridine nitrogen. Additional product ions at *m*/*z* 273.06547 (C_17_H_9_N_2_O_2_^+^), 245.07061 (C_16_H_9_N_2_O^+^), and 217.07588 (C_15_H_9_N_2_^+^) are consistent with successive cleavages within the aromatic scaffold (Figures S16 and S34).

The ^1^H NMR spectrum of DP-3 (Figure S17, Table S5) showed noticeable chemical shift changes in the pyridine ring region when compared to PER. In particular, the signal assigned to H-24 shifted from δ 8.59 ppm in PER to δ 8.29 ppm in DP-3, reflecting the altered electronic environment caused by N-oxidation. Additionally, an increased separation between the doublets corresponding to H-8 and H-10 of the pyridone ring was observed, indicating perturbation of the conjugated system.

The ^13^C NMR spectrum of DP-3 (Figure S18, Table S5) further supported this assignment. Significant changes were observed for carbons associated with the pyridine and pyridone rings, including C-24 (δ 140.46 ppm), C-8 (δ 140.52 ppm), C-10 (δ 141.01 ppm), and C-9 (δ 110.28 ppm). These shifts reflect the altered electron density distribution induced by *N*-oxidation while preserving the overall skeleton of PER.

The COSY spectrum (Figure S19) confirmed that the proton–proton coupling networks within the aromatic systems were preserved, indicating that no bond cleavage occurred in these regions. The HSQC spectrum (Figure S20) enabled the direct correlation of protons with their attached carbons, validating the revised assignments and confirming that the structural changes were localized to the pyridine moiety.

These findings are consistent with previously reported oxidative degradation of PER to its pyridine *N*-oxide derivative, DP-3 (2-(2-oxo-5-(1-oxopyridin-2-yl)-1-phenyl-1,2-dihydropyridin-3-yl) benzonitrile).^9,11^ Under oxidative conditions, degradation of PER occurs primarily through oxidation of the pyridine moiety, involving the interaction of peroxide species with the pyridine nitrogen to form the corresponding *N*-oxide. Such transformations are well documented for nitrogen-containing heterocycles exposed to hydrogen peroxide. The proposed structure of DP-3 is supported by combined HRMS/MS and NMR data.

#### 3.4.3 Alkaline Hydrolysis

DP-4 showed [M + H]^+^ at *m*/*z* 265.09696 (C_16_H_13_N_2_O_2_^+^) (Table S1). The MS/MS spectrum (Figure S21) exhibited a fragment at *m*/*z* 247.08651 (−18 Da), consistent with dehydration and supporting the presence of a hydroxylated species. Further product ions at *m*/*z* 237.10233 (C_15_H_13_N_2_O^+^), consistent with loss of a small neutral CO, and 220.07568 (C_15_H_10_NO^+^) can also be observed (Figure S35).

The ^1^H NMR spectrum of DP-4 (Figure S22, Table S6) showed a significantly simplified pattern when compared to PER, indicating the loss of one of the heterocyclic subunits. In particular, the absence of characteristic pyridone and benzyl protons observed in PER confirms that the original heterocyclic framework was disrupted. Two highly deshielded singlets were observed at δ 12.15 ppm (H-20) and δ 10.11 ppm (H-10), which are characteristic of exchangeable protons and are consistent with the presence of hydroxyl and enolic-type functionalities, due to the addition of a hydroxyl group at C-8. Importantly, none of these highly deshielded protons exhibited correlations in the COSY spectrum (Figure S24), indicating that they are isolated and not part of a proton-coupled spin system.

The ^13^C NMR spectrum of DP-4 (Figure S23, Table S6) showed a diagnostic carbonyl carbon signal at δ 184.12 ppm (C-8), which is significantly downfield compared to the pyridone carbonyl of PER. This chemical shift is characteristic of conjugated ketone or enone systems and is consistent with the proposed ring-cleavage and rearrangement process. The remaining aromatic carbon signals corresponding to the benzonitrile and pyridine moieties were preserved, indicating that these substructures remained intact.

The COSY and HSQC spectra (Figures S24 and S25) confirmed the preservation of the benzonitrile and pyridine proton–carbon frameworks, while clearly demonstrating the absence of correlations associated with the original pyridone ring. This selective loss of correlations is consistent with a cleavage of the pyridone moiety under alkaline conditions.

Taken together, HRMS data, the characteristic dehydration behavior observed in MS/MS spectra, the presence of an additional highly deshielded carbonyl carbon, and the altered COSY and HSQC correlation patterns provide convergent evidence for the proposed structure of DP-4. These findings support its identification as 2-((2*E*)-3-hydroxy-1-oxo-4-(pyridin-2-yl)­but-2-en-2-yl)­benzonitrile, a previously unreported ring-opened degradation product formed under alkaline hydrolysis.

Under alkaline conditions, degradation of PER is driven by nucleophilic attack at electrophilic centers within the conjugated system, leading to structural rearrangements and ring–opening reactions. In this context, DP-4 is proposed to arise from nucleophilic attack followed by cleavage of the pyridone ring, generating a more open-chain structure.

DP-5, formed under alkaline hydrolysis, showed a protonated ion at *m*/*z* 366.12309 (C_23_H_16_N_3_O_2_^+^) (Table S1), isomeric with DP-3 but chromatographically distinct. Unlike DP-3, its MS/MS spectrum showed a neutral loss of 18 Da (H_2_O), supporting hydroxylation rather than N-oxidation. Additional fragments at *m*/*z* 322.1335 (C_22_H_16_N_3_^+^) and 260.0942 (C_17_H_12_N_2_O^+^) are consistent with further cleavage of the oxygenated aromatic scaffold, and low-mass ions at *m*/*z* 124.0393 (C_6_H_6_NO_2_^+^) and 96.0447 (C_5_H_6_NO^+^) were also observed (Figures S26 and S36).

The ^1^H NMR spectrum of DP-5 (Figure S27, Table S7) showed marked differences compared to PER. Notably, the proton H-8, which is clearly observed in the spectrum of PER, was absent in DP-5. This disappearance indicates substitution at this position, consistent with hydroxylation. Additionally, significant chemical shift changes were observed for the protons of the pyridone ring, reflecting perturbation of the electronic environment caused by the newly introduced hydroxyl group. Although DP-5 is proposed as a hydroxylated derivative, the −OH proton (H-28) was not observed in the ^1^H NMR spectrum, most likely due to rapid exchange with the deuterated solvent and line broadening effects commonly observed for labile hydroxyl protons.

The COSY spectrum (Figure S29) showed that the pyridone proton H-10 no longer correlated with neighboring protons, indicating a disruption of the original spin system. This loss of correlation further supports a substitution at the adjacent position (C-8), which interrupts the original coupling network.

The ^13^C NMR spectrum of DP-5 (Figure S28, Table S7) showed a new highly deshielded signal at δ 188.11 ppm, which is characteristic of a conjugated carbonyl group perturbed by adjacent hydroxyl substitution. This shift is consistent with hydroxylation at C-8 of the pyridone ring, which strongly modifies the local electronic environment. The HSQC spectrum (Figure S30) confirmed the remaining proton–carbon correlations and supported the assignment of the preserved substructures.

The combined analysis of HRMS data, MS/MS fragmentation patterns, the disappearance of the H-8 signal, the altered COSY correlations, and the appearance of a new downfield carbonyl carbon signal provides strong and convergent evidence for the identification of DP-5 as a hydroxylated derivative of PER. These findings support its assignment as 2-(4-hydroxy-2-oxo-1-phenyl-5-(pyridin-2-yl)-1,2-dihydropyridin-3-yl)­benzonitrile, formed by hydroxylation at C-8 of the pyridone ring. To the best of our knowledge, this DP has not been previously reported. The formation of DP-5 is attributed to hydroxylation of the aromatic system, likely mediated by reactive oxygen species under the applied conditions.

Finally, under alkaline conditions, an additional major peak (RT ≈ 14 min; Figure 1) was observed. HRMS analysis revealed a protonated molecular ion identical to that of PER, indicating that no change in molecular formula occurred. Moreover, MS/MS data were not sufficiently informative to support structural modification.

NMR analyses (1D and 2D) showed that the main proton spin systems were preserved, suggesting that the molecular framework remains unchanged. However, chemical shift variations were observed, indicating changes in the electronic environment.

Considering these findings, this species is more consistent with a reversible transformation, such as base-promoted tautomeric or electronic rearrangement, rather than a classical degradation process.^36^ Therefore, it was not classified as a degradation product.

### 3.5 Accelerated Stability Studies of PER Tablets

The accelerated stability study was designed to assess the practical relevance of the degradation pathways identified under forced degradation. Prototype PER tablets containing excipients with acidic (tartaric acid), alkaline (sodium carbonate), or oxidative (urea–hydrogen peroxide) properties were stored under accelerated conditions (40 °C/75% RH) for up to 24 months. A single degradation product was detected in tablets formulated with the acidic excipient after 12 months, corresponding to DP-1 (Figure 3), in both semipermeable and impermeable packaging. Considering that the study conditions followed an accelerated stability protocol, this finding suggests that DP-1 is a relevant degradation product under long-term storage conditions and may be expected to form during the shelf life of PER tablets. The presence of DP-1 became more evident over storage time, indicating its formation under accelerated conditions, likely influenced by the excipient environment. These observations are qualitative and based on chromatographic signal trends, and therefore do not represent absolute quantification.

**Figure 3:**
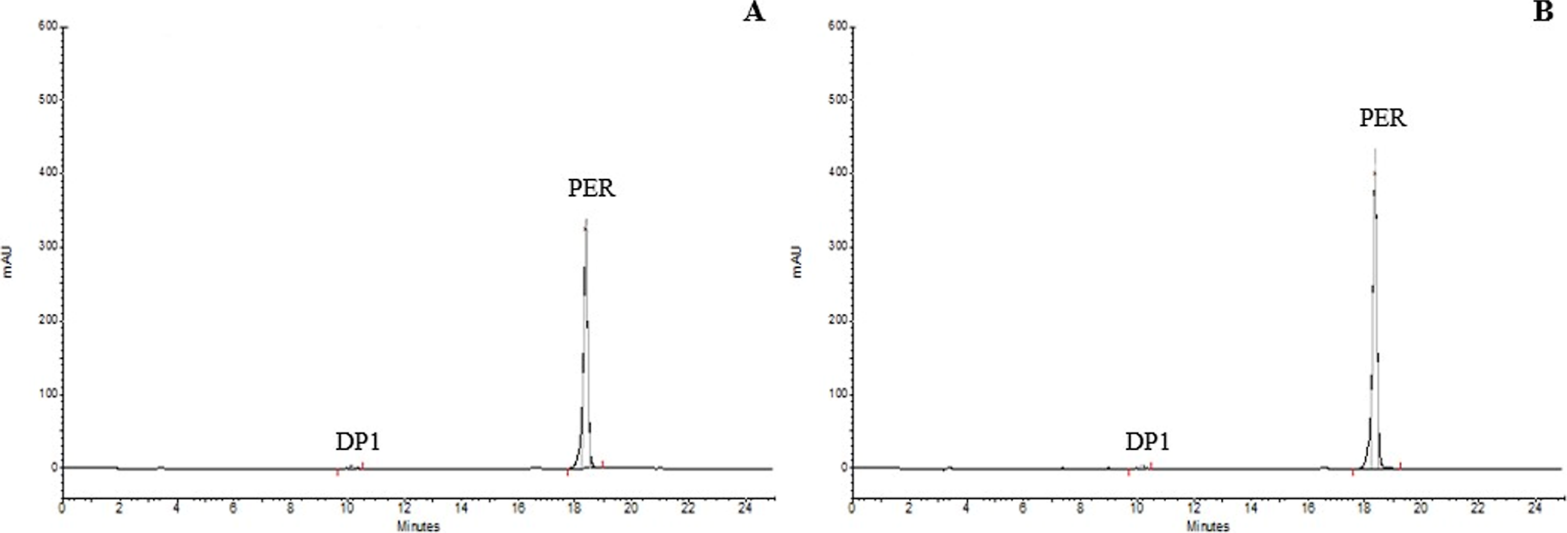
Accelerated stability chromatograms of tablets containing acid excipient in semipermeable (A) and impermeable (B) packaging.

In addition, this study demonstrated that for development of new formulations with PER is important to avoid acidic excipients, since is possible the formation of degradation product DP-1. No detectable degradation was observed in tablets containing alkaline or oxidative excipients throughout the study period.

### 3.6 Prediction of In Silico Toxicity Properties of Perampanel DPs in Comparison with PER

The ICH M7 guideline establishes a framework for the classification of impurities according to their mutagenic and carcinogenic potential and defines appropriate control strategies. All PER degradation products (DPs) were evaluated using two complementary in silico approaches: a statistical-based model (Method I) and an expert rule–based system (Method II). This dual-model strategy was intentionally adopted in accordance with ICH M7 recommendations, which emphasize the combined use of statistical and expert knowledge–based QSAR approaches to improve confidence in mutagenicity predictions. No structural alerts associated with DNA reactivity were identified for any DP using either methodology (Table S8). Although Method II indicated that some compounds were outside the applicability domain, an expert review and a third independent software evaluation were performed to support the predictions. This additional assessment step ensured a conservative and scientifically robust interpretation of the in silico results, minimizing uncertainty associated with domain limitations. Accordingly, none of the DPs exhibited mutagenic concern, allowing their classification as ICH M7 Class 4 or Class 5 impurities, i.e., nonmutagenic substances.^13^

Beyond mutagenicity assessment, additional in silico toxicity end points were evaluated to provide a broader toxicological profile of PER and its degradation products, as described in Section 2.8. With respect to general toxicity, PER and its DPs were further evaluated for potential liver injury. Hepatotoxicity associated with antiepileptic drugs is known to vary widely across this therapeutic class.^37^ In silico predictions indicated increased probabilities for elevations of liver enzymes, namely aspartate aminotransferase (AST), alanine aminotransferase (ALT), gamma-glutamyl transferase (GGT), and alkaline phosphatase (ALP), for some DPs, with the exception of DP-4. Hepatotoxic potential was considered when simultaneous elevations of AST and ALT were predicted, while concomitant increases in GGT or ALP were interpreted as supportive indicators of liver injury. Although such events are uncommon, sporadic clinical reports of PER-associated liver injury have been described, warranting continued monitoring.^38^

Blockade of the human ether-à-go-go–related gene (hERG) potassium channel is associated with QT interval prolongation and an increased risk of cardiac arrhythmias. In this study, a low cardiotoxicity risk was predicted for PER and all DPs, in agreement with clinical data showing no clinically relevant QT interval prolongation.^39,40^

Finally, acute oral toxicity was predicted in rats and classified according to the Globally Harmonized System (GHS). The estimated LD_50_ values for PER DPs ranged from 940 to 2500 mg/kg. DP-1 and DP-3 were classified as low-risk compounds (Class 5, LD_50_ > 2000 mg/kg), whereas DP-2, DP-4, and DP-5 were assigned to Class 4 (300 < LD_50_ ≤ 2000 mg/kg), still indicating low acute toxicity. These findings indicate that, although some degradation products present moderate predicted acute toxicity, their overall toxicological profiles remain consistent with low regulatory concern.

## 4 Conclusions

A stability-indicating HPLC–UV (DAD) method was developed and successfully applied to investigate the degradation behavior of PER. In addition to confirming previously reported degradation products (DP-1, DP-2, and DP-3), two novel degradation products (DP-4 and DP-5) were isolated and structurally characterized, enabling a more comprehensive understanding of the PER degradation profile. Importantly, DP-1 was identified during accelerated stability studies of tablets formulated with acidic excipients, demonstrating an incompatibility between PER and acidic formulation components. These findings highlight the need to avoid acidic excipients during the development of solid dosage forms containing PER.

The in silico toxicological assessment, conducted using complementary statistical and expert-rule methodologies validated according to OECD principles, supports the regulatory relevance of the generated data. All PER degradation products were classified as ICH M7 Class 4 or Class 5, indicating no mutagenic concern. To address limitations related to applicability domain, an additional software platform was incorporated to strengthen expert evaluation. While in silico predictions suggested a potential risk of hepatotoxicity for some degradation products, these findings should be interpreted cautiously and warrant further investigation. In contrast, a consistently low cardiotoxicity risk was predicted for PER and all DPs. Overall, the combined analytical, stability, and toxicological data provide a scientifically robust basis for understanding the degradation behavior of PER and support informed decisions in formulation development and impurity risk assessment.

## Supplementary Material



## Notes

HRMS/MS fragmentation data and proposed structural assignments, MS/MS spectra, ^1^H and ^13^C NMR spectra, COSY and HSQC spectra; in silico toxicity evaluation, proposed mass fragmentation pathways and UV absorption spectra of PER and its degradation products (DP-1 to DP-5) (PDF)
