# Supplementary material for: Identification and Characterization of Perampanel Degradation Products: Stability-Indicating HPLC and In Silico Toxicological Assessment
Source: ACS Omega. 2026 Mar 14;11(11):18115–24. doi: 10.1021/acsomega.5c13204 (PMC13019199; doi:10.1021/acsomega.5c13204)
Supplement: Supplementary file 1 [file ao5c13204_si_001.pdf]

Supporting Information

Identification and Characterization of Perampanel Degradation Products: Stability-Indicating HPLC and *In Silico* Toxicological Assessment

Jéssica Domingos da Silva,[a] Gil Mendes Viana,[b]* Luana Gonçalves de Souza,[a] Bárbara Abrahim-Vieira,[c] Alessandra Mendonça Teles de Souza,[c] Carina de Souza Anselmo[d], Henrique Marcelo Gualberto Pereira [d], Lucio Mendes Cabral,[b] Valéria Pereira de Sousa[a]

[a]*Universidade Federal do Rio de Janeiro, Faculdade de Farmácia, Laboratório de Controle de Qualidade, Rio de Janeiro, RJ, Brazil.*

[b]*Universidade Federal do Rio de Janeiro, Faculdade de Farmácia, Laboratório de Tecnologia Industrial Farmacêutica, Rio de Janeiro, RJ, Brazil.*

[c]*Universidade Federal do Rio de Janeiro, Faculdade de Farmácia, Laboratório de Modelagem Molecular & QSAR (ModMolQSAR), Rio de Janeiro, RJ, Brazil.*

[d]*Universidade Federal do Rio de Janeiro, Instituto de Química, Laboratório Brasileiro de Controle de Dopagem LBCD – LADETEC, Rio de Janeiro, RJ, Brazil.*

*Corresponding author: gmviana@farmacia.ufrj.br

Table S1. HRMS/MS fragmentation data and proposed structural assignments for PER and its degradation products.

PER and DPs	Molecular formula $[M+H]^+$	Experimental mass (*m/z*)	Theoretical mass (*m/z*)	Error (ppm)	RDB	Neutral loss / fragmentation	Proposed structural assignment
**PER**	$C_{23}H_{16}N_3O$	350.12827	350.12879	-1.49	17.5	-	Protonated molecular ion
	$C_{16}H_{11}N_2O$	247.08630	247.08659	-1.57	12.5	Cleavage of benzonitrile moiety	Complementary fragment retaining the pyridone–aryl/pyridyl core
	$C_{15}H_{11}N_2$	219.09134	219.09167	-1.53	11.5	-CO (28 Da)	Loss of carbonyl group from pyridone-containing fragment
	C_7H_6N	104.04972	104.04948	2.35	5.5	Aromatic cleavage	Diagnostic benzonitrile-related fragment
**DP1**	$C_{23}H_{18}N_3O_2$	368.13943	368.13935	0.21	16.5	—	Protonated molecular ion
	$C_{23}H_{15}N_2O_2$	351.11213	351.11280	-1.92	17.5	$-NH_3$ (17 Da)	Loss of ammonia, consistent with amide functionality
	$C_{16}H_{10}NO_2$	248.07013	248.07061	-1.93	12.5	Aromatic cleavage	Fragment retaining oxygenated aromatic core
	$C_{15}H_{10}NO$	220.07569	220.07569	-1.13	11.5	-CO (28 Da)	Carbonyl-containing aromatic fragment
	C_7H_6N	104.04954	104.04948	0.65	5.5	Aromatic cleavage	Diagnostic benzonitrile-related fragment
**DP2**	$C_{23}H_{17}N_2O_3$	369.12270	369.12337	-1.80	16.5	—	Protonated molecular ion
	$C_{23}H_{15}N_2O_2$	351.11197	351.11280	-2.38	17.5	$-H_2O$ (18 Da)	Dehydration, consistent with carboxylic acid derivative
	$C_{22}H_{15}N_2O$	323.11687	323.11789	-3.17	16.5	-CO (28 Da)	Carbonyl-containing fragment
	$C_{16}H_{10}NO_2$	248.06979	248.07061	-3.27	12.5	Aromatic cleavage	Fragment retaining oxygenated aromatic core
	$C_{15}H_{10}NO$	220.07497	220.07569	-3.25	11.5	-CO (28 Da)	Carbonyl-containing aromatic fragment
	C_7H_6NO	104.04941	104.04948	-0.63	5.5	Aromatic cleavage	Diagnostic benzonitrile-related fragment

**DP3**	$C_{23}H_{16}N_3O_2$	366.12324	366.12370	-1.27	17.5	—	Protonated molecular ion
	$C_{23}H_{15}N_3O$	349.12041	349.12096	-1.58	18.0	-17 Da	Characteristic neutral loss of protonated N-oxides
	$C_{22}H_{16}N_3$	322.13306	322.13387	-2.54	16.5	Small neutral loss	Further fragmentation of aromatic core
	$C_{17}H_9N_2O_2$	273.06547	273.06585	-1.41	14.5	Aromatic cleavage	Oxygenated aromatic fragment
	$C_{16}H_9N_2O$	245.07061	245.07094	-1.36	13.5	Aromatic cleavage	Oxygenated aromatic fragment
	$C_{15}H_9N_2$	217.07588	217.07602	-0.66	12.5	Aromatic cleavage	Aromatic scaffold fragment
**DP4**	$C_{16}H_{13}N_2O_2$	265.09696	265.09715	-0.72	11.5	—	Protonated molecular ion
	$C_{16}H_{11}N_2O$	247.08651	247.08659	-0.33	12.5	-H_2O (18 Da)	Dehydration of hydroxylated fragment
	$C_{15}H_{13}N_2O$	237.10233	237.10224	0.36	10.5	- CO (28 Da)	Loss of carbonyl group from core
	$C_{15}H_{10}NO$	220.07568	220.07569	-0.06	11.5	Small neutral loss	Oxygenated fragment
**DP5**	$C_{23}H_{16}N_3O_2$	366.12309	366.12370	-1.67	17.5	—	Protonated molecular ion
	$C_{23}H_{14}N_3O$	348.11266	348.11314	-1.37	18.5	-H_2O (18 Da)	Dehydration, consistent with hydroxylation
	$C_{22}H_{16}N_3$	322.13347	322.13387	-1.25	16.5	Aromatic cleavage	Cleavage of aromatic core
	$C_{17}H_{12}N_2O$	260.09417	260.09441	-0.95	13.0	Aromatic cleavage	Oxygenated aromatic fragment
	$C_6H_6NO_2$	124.03930	124.03930	-0.06	4.5	Deep fragmentation	Low-mass oxygenated aromatic ion
	C_5H_6NO	96.04466	96.04439	2.86	3.5	Deep fragmentation	Low-mass nitrogen-containing aromatic ion

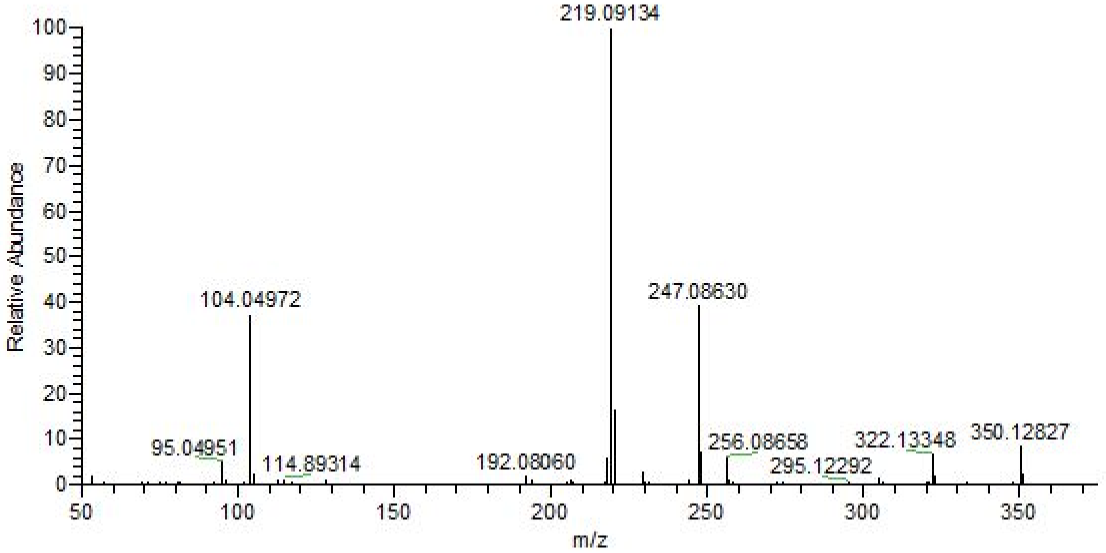

Figure S1. MS/MS spectrum of Perampanel.

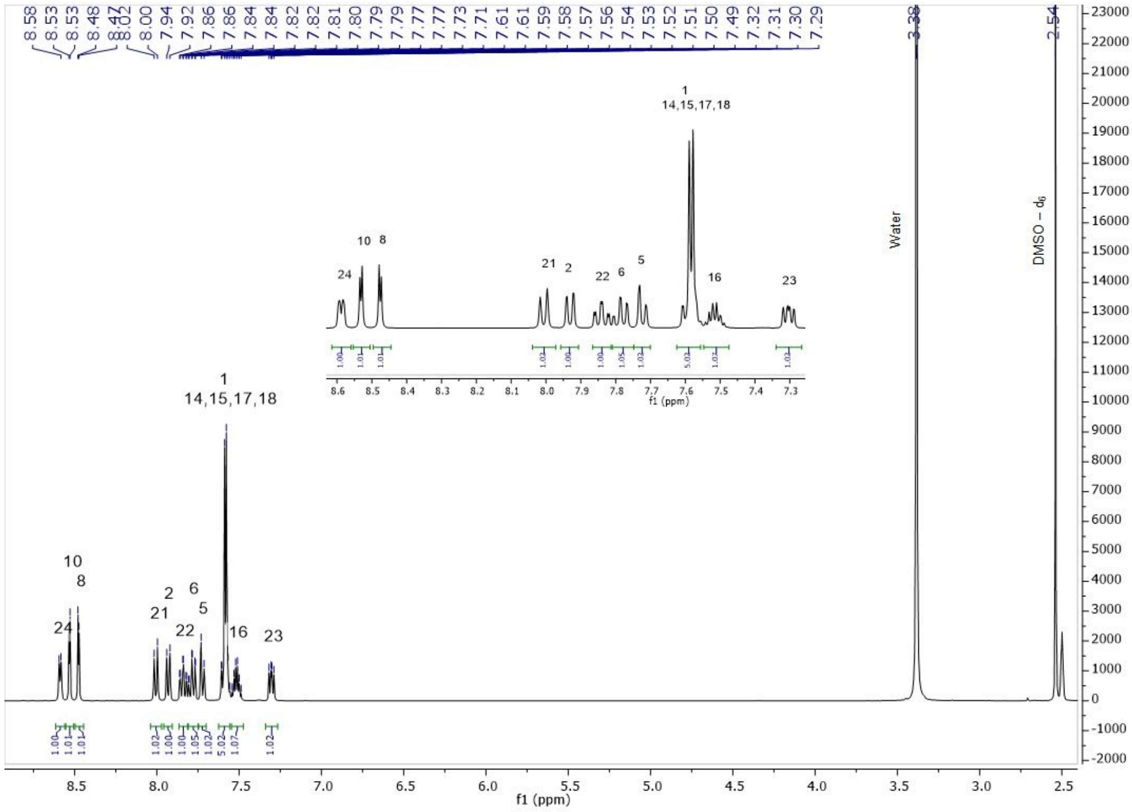

Figure S2. ^{1}H NMR spectrum of Perampanel in DMSO-d_6.

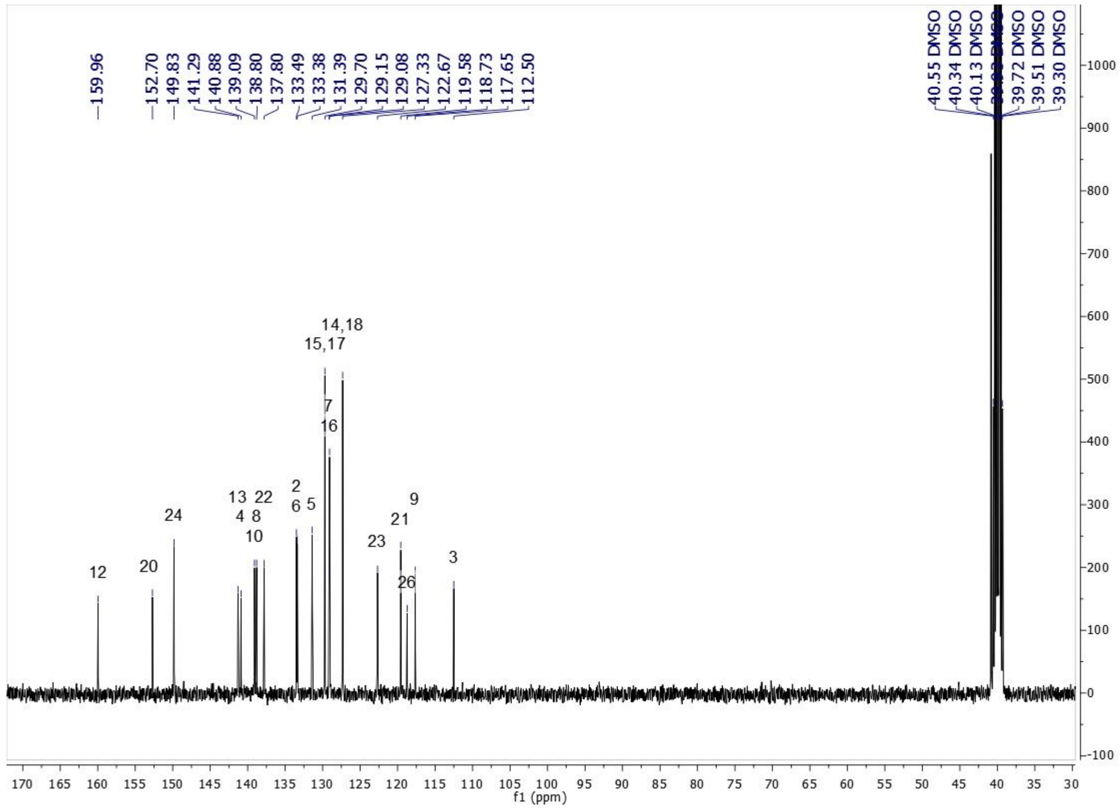

Figure S3. ^{13}C NMR spectrum of Perampanel in DMSO-d_6.

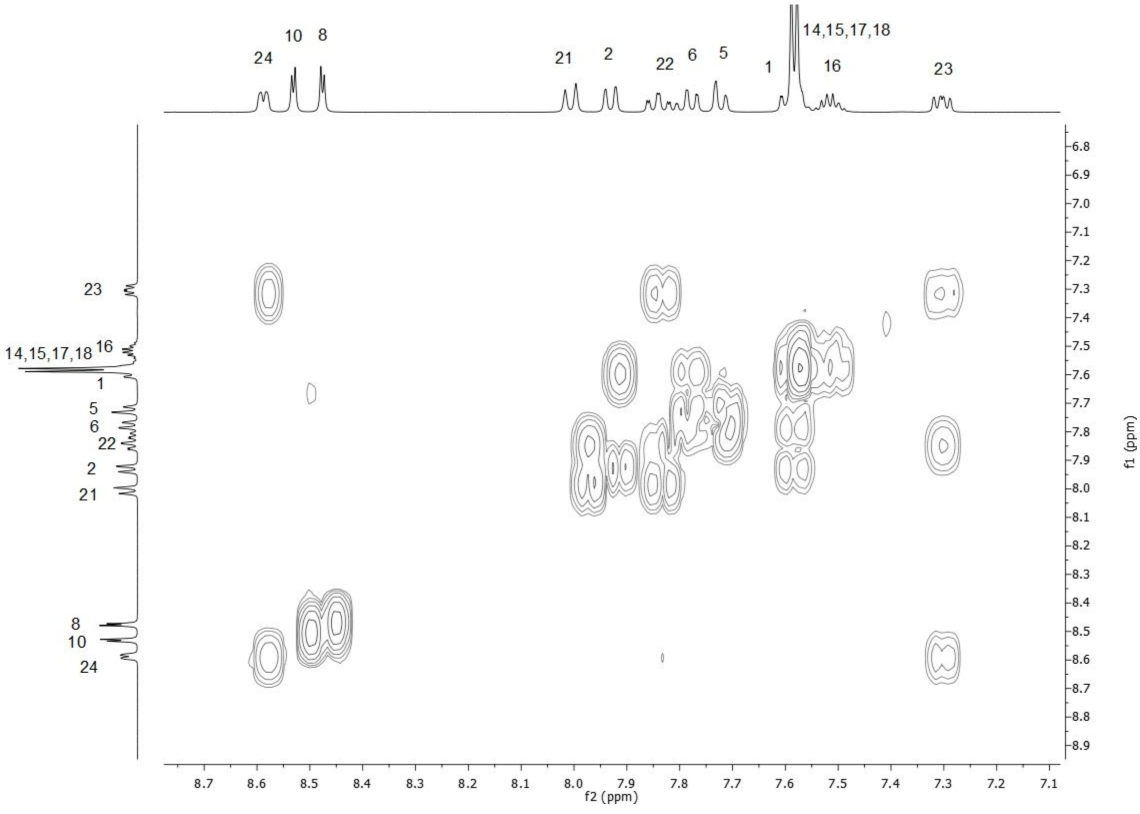

Figure S4. COSY spectrum of Perampanel in DMSO-d_6.

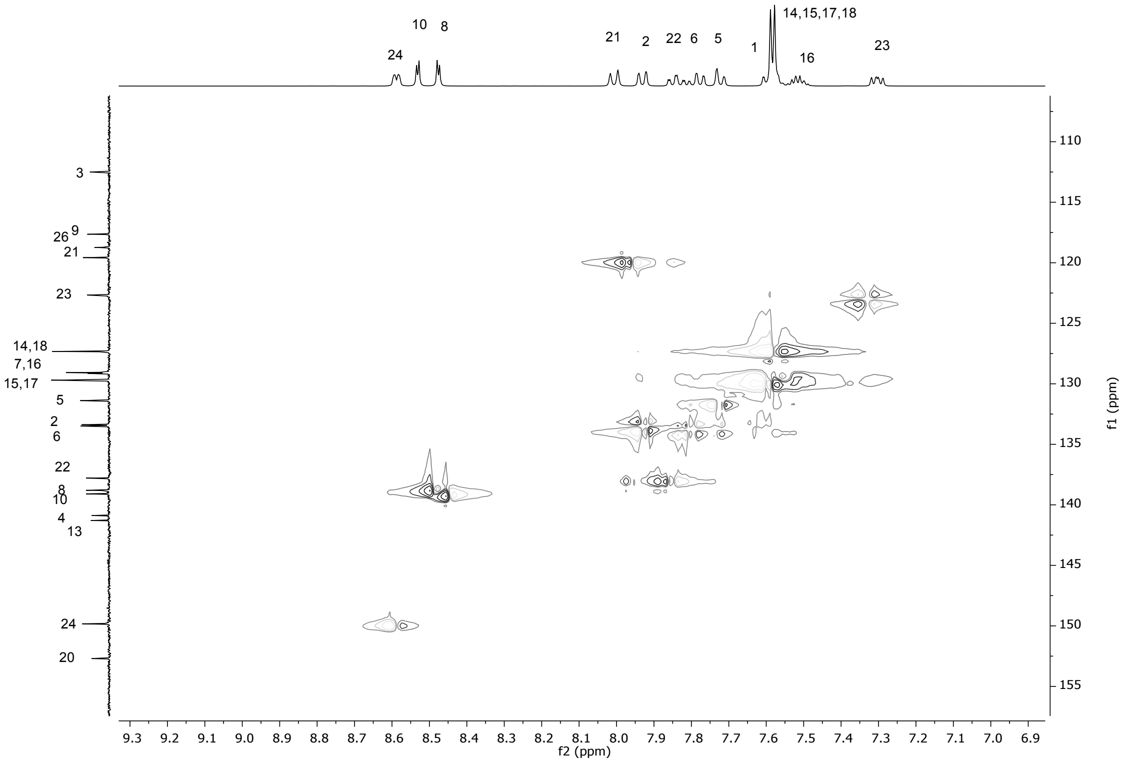

Figure S5. HSQC spectrum of Perampanel in DMSO-d_6.

Table S2. ^{1}H, ^{13}C, COSY and HSQC data of Perampanel in DMSO-d_6.

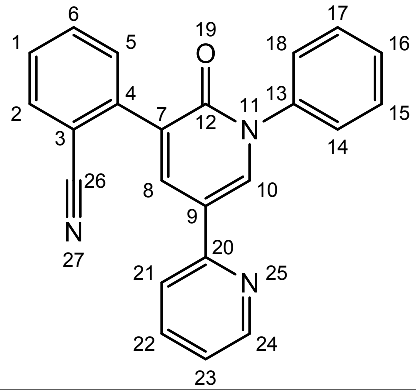

Position	^{1}H (δ ppm)	^{13}C (δ ppm)	COSY	HSQC
1	7.63-7.55(m)	-	H-2, H-6	-
2	7.93 (d)	133.49	H-1	C-2
3	-	112.50	-	-
4	-	140.88	-	-
5	7.72 (d)	131.39	H-6	C-5
6	7.79 (d)	133.38	H-1, H-5	C-6
7	-	129.15	-	-
8	8.48 (d)	138.80	H-10	C-8
9	-	117.65	-	-
10	8.53 (d)	139.09	H-8	C-10
12	-	159.96	-	-
13	-	141.29	-	-
14/18	7.63-7.55(m)	127.33	H-15/ H-17, H-16	C-14/C-18
15/17	7.63-7.55(m)	129.70	H-14/H-18, H-16	C-15/C-17
16	7.52 (m)	129.08	H-14/H18, H-15/H-17	C-16
20	-	152.70	-	-
21	8.01(d)	119.58	H-22	C-21
22	7.84 (td)	137.80	H-21, H-23	C-22
23	7.30 (dd)	122.67	H-22, H-24	C-23
24	8.59 (d)	149.83	H-23	C-24
26	-	118.73	-	-

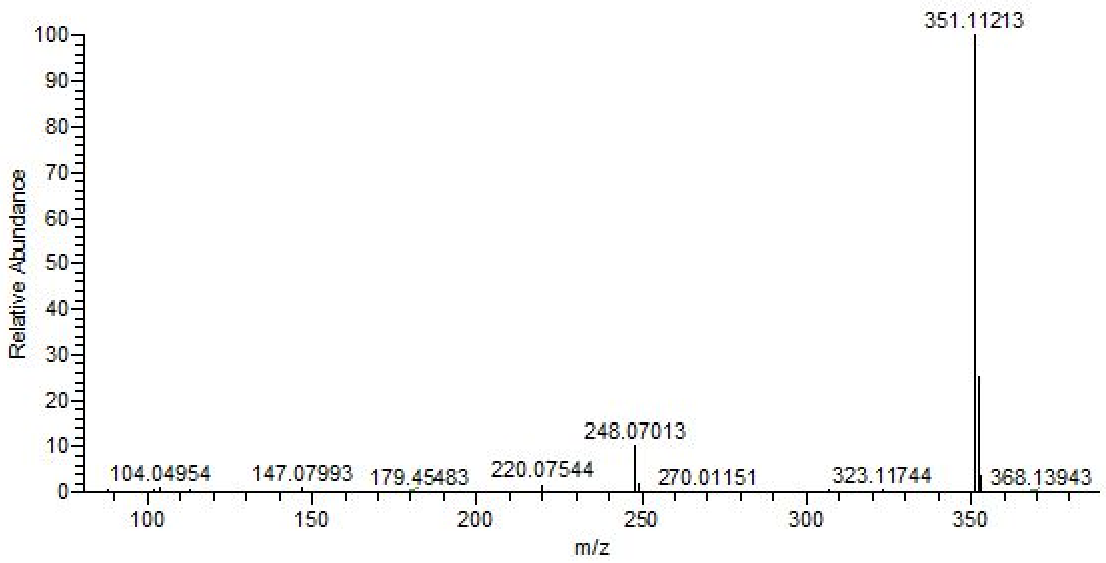

Figure S6. MS/MS spectrum of DP-1.

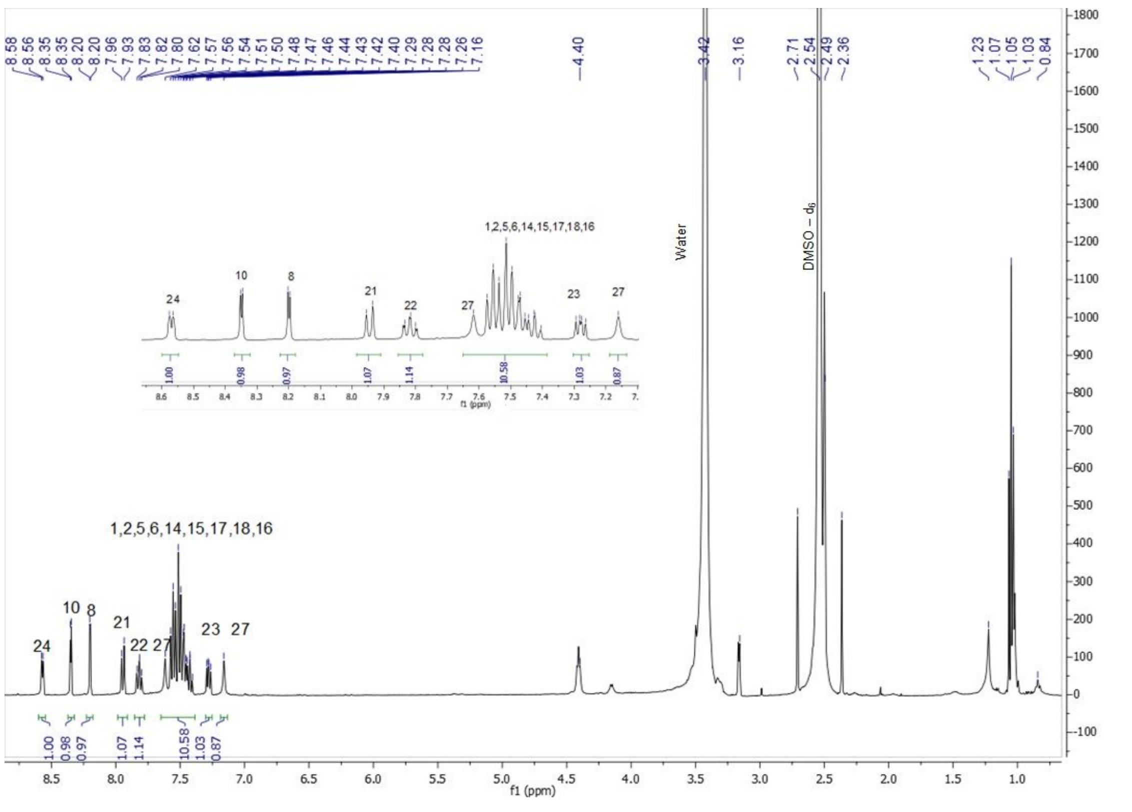

Figure S7. ^{1}H NMR spectrum of DP-1 in DMSO-d_6.

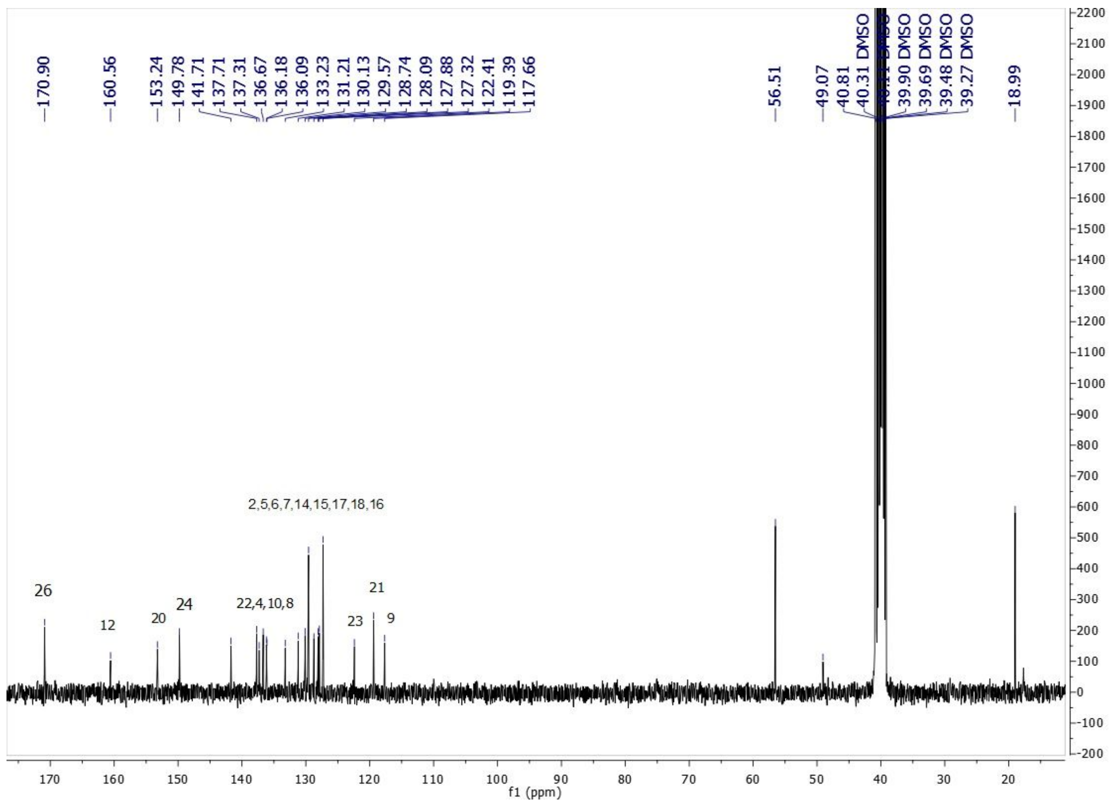

Figure S8. ^{13}C NMR spectrum of DP-1 in DMSO-d_6.

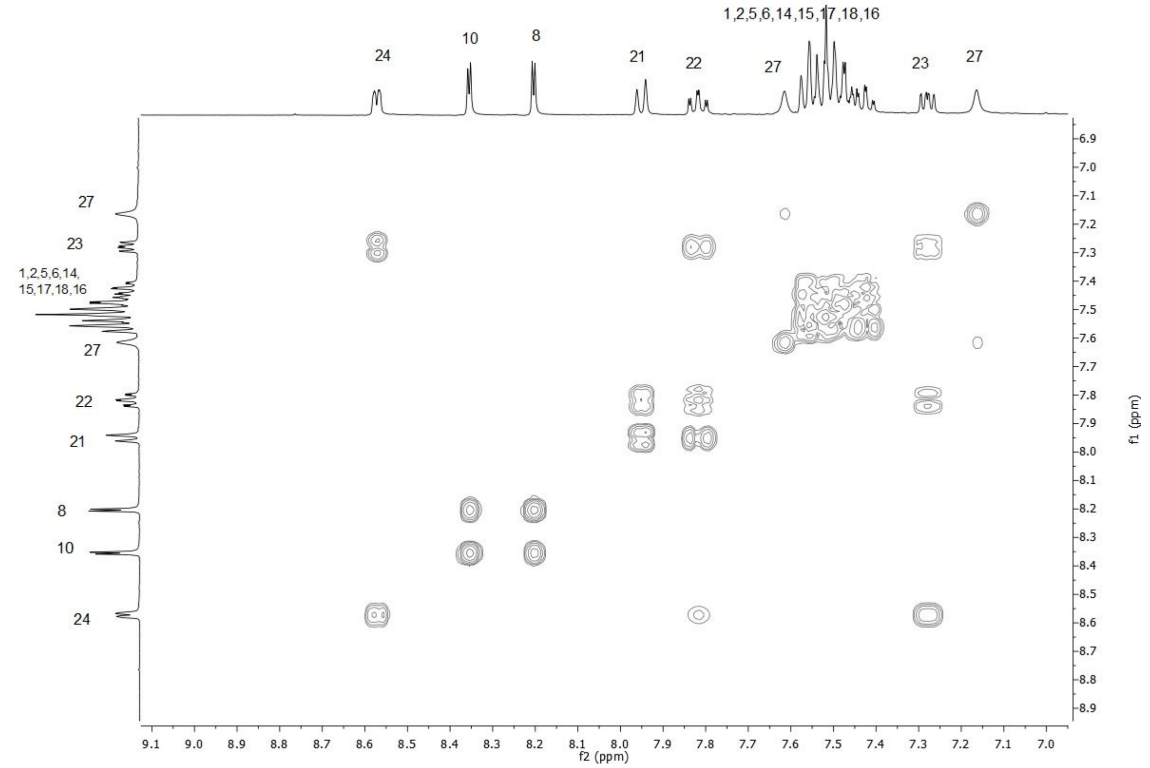

Figure S9. COSY NMR spectrum of DP-1 in DMSO-d_6.

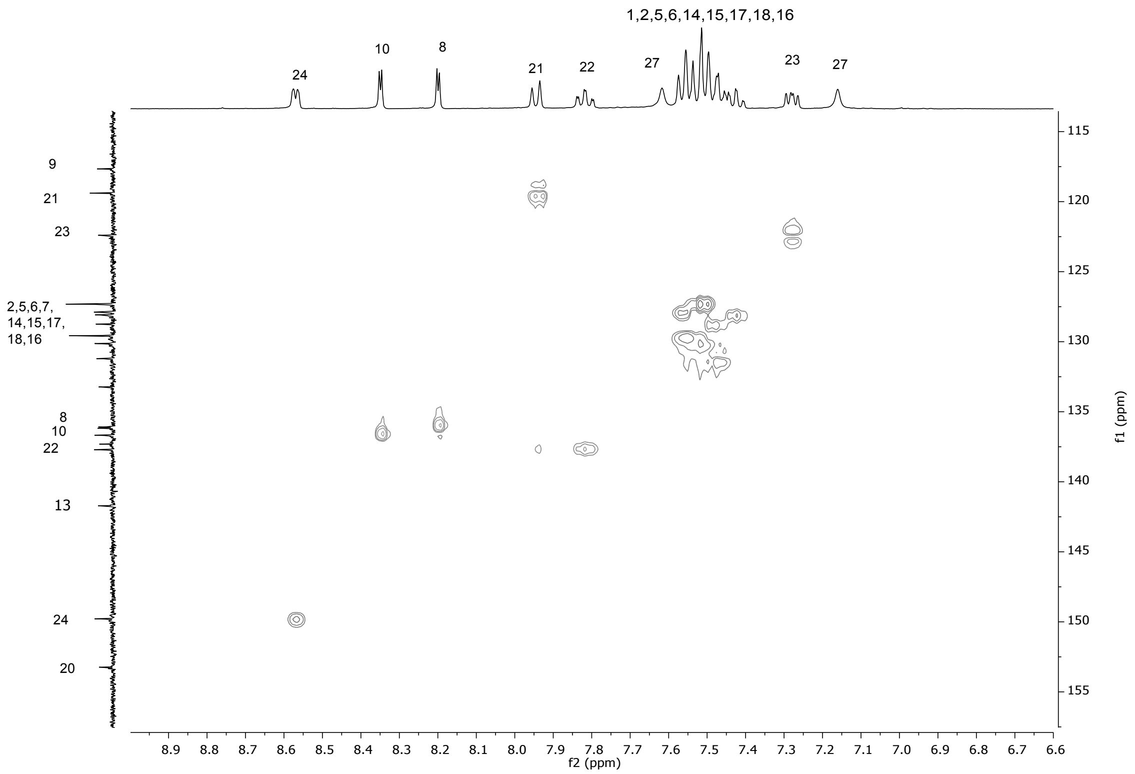

Figure S10. HSQC NMR spectrum of DP-1 in DMSO-d_6.

Table S3. ^{1}H, ^{13}C, COSY and HSQC data of DP-1 in DMSO-d_6.

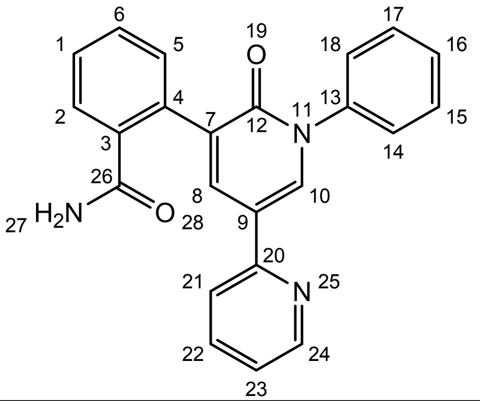

Position	^{1}H (δ ppm)	^{13}C (δ ppm)	COSY	HSQC
1	7.65 – 7.39 (m)	-	H-1, H-2, H-5, H-6, H-14/H-18, H-15/H-17, H-16	-
2,5,6,7,14,18, 15,17,16	7.65 – 7.39 (m)	133.23, 131.21, 130.13, 128.74, 128.09, 127.88, 127.32	H-1, H-2, H-5, H-6, H-14/H-18, H-15/H-17, H-16	C-2, C-5, C-6, C-7, C-14/C-18, C-15/C-17, C-16
3	-	-	-	-
4	-	137.31	-	-
8	8.20 (d)	136.13	H-10	C-8
9	-	117.66	-	-
10	8.35 (d)	136.67	H-8	C-10
12	-	160.56	-	-
13	-	141.71	-	-
20	-	153.24	-	-
21	7.94 (d)	119.39	H-22	C-21
22	7.82 (d)	137.71	H-21, H-23, H-24	C-22
23	7.28 (dd)	122.41	H-22, H-24	C-23
24	8.57 (d)	149.78	H-22, H-23	C-24
26	-	170.90	-	-
27	7.65 – 7.39 (m), 7.16 (s)	-	H-27	-

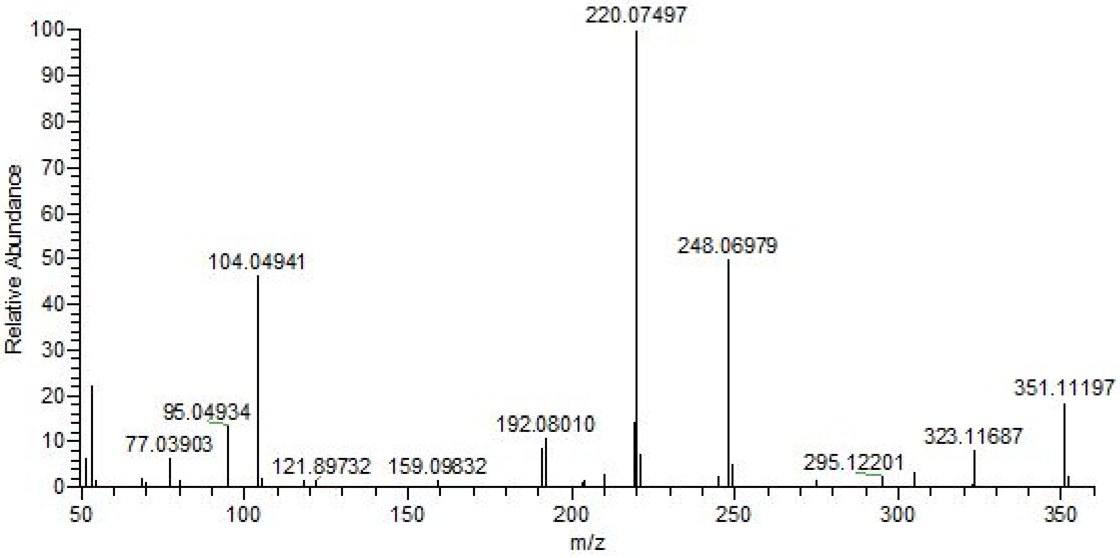

Figure S11. MS/MS spectrum of DP-2.

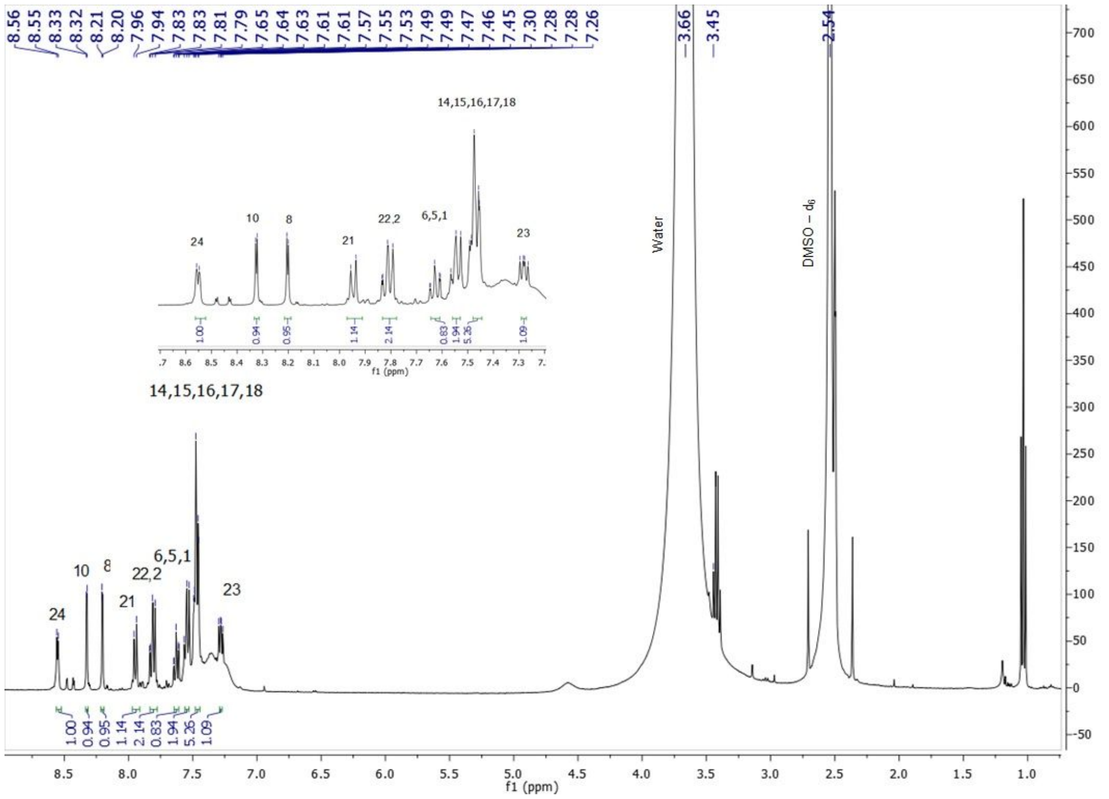

Figure S12. ^{1}H NMR spectrum of DP-2 in DMSO-d_6.

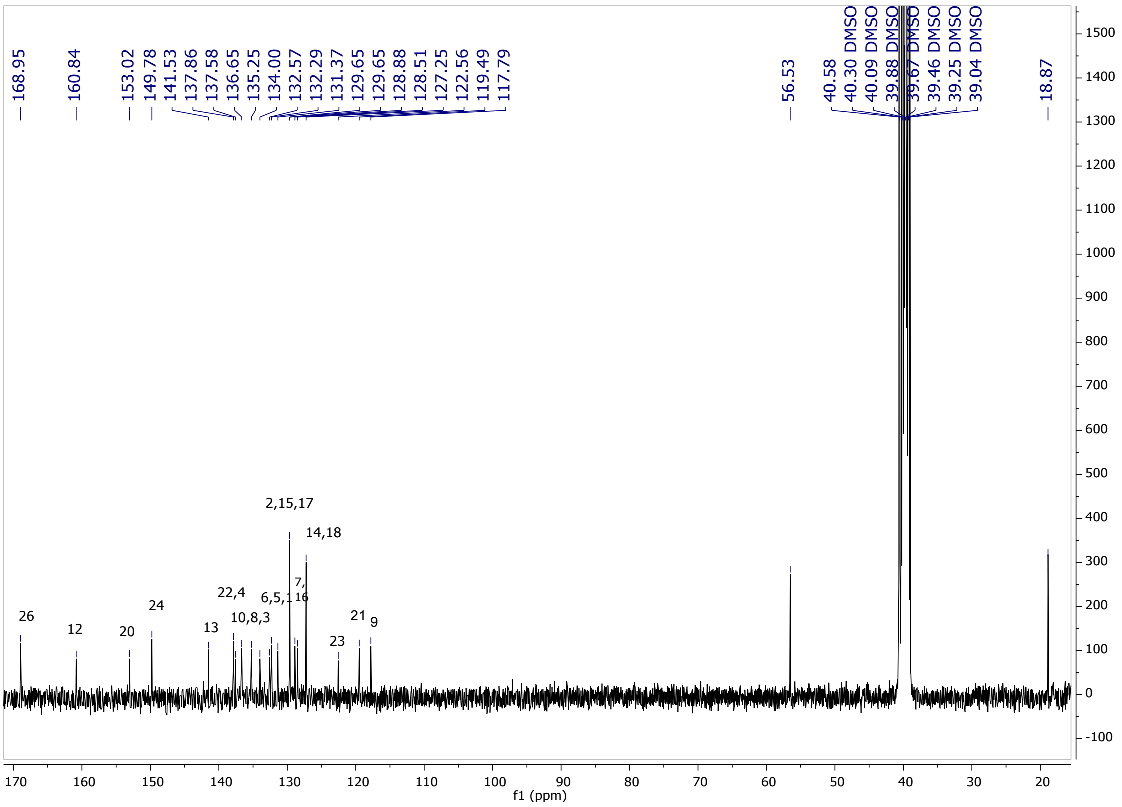

Figure S13. ^{13}C NMR spectrum of DP-2 in DMSO-d_6.

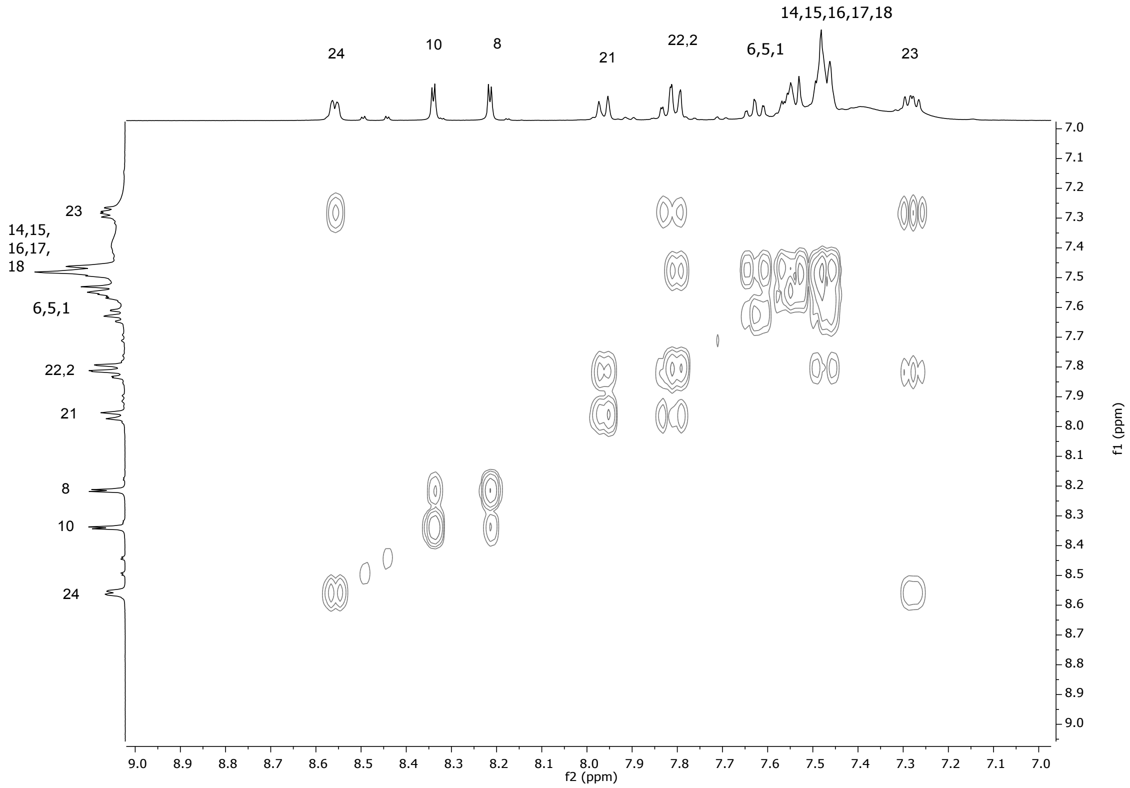

Figure S14. COSY NMR spectrum of DP-2 in DMSO-d_6.

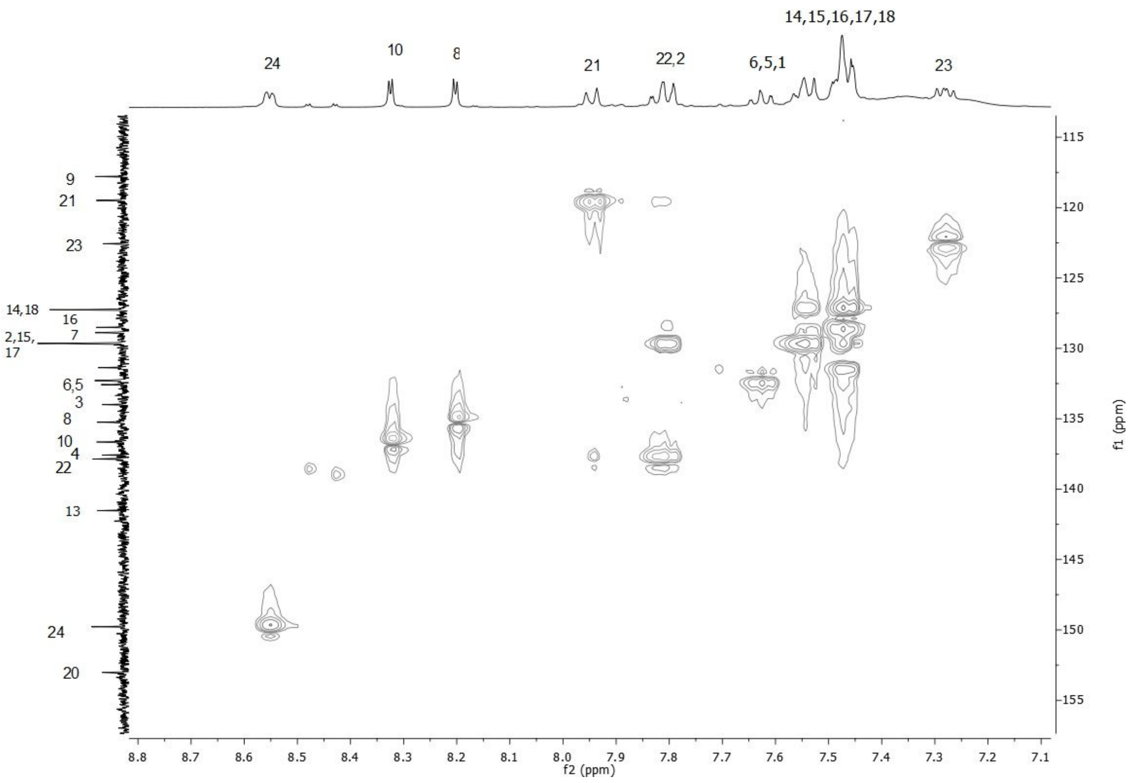

Figure S15. HSQC NMR spectrum of DP-2 in DMSO-d_6.

Table S4. ^{1}H, ^{13}C, COSY and HSQC data of DP-2 in DMSO-d_6.

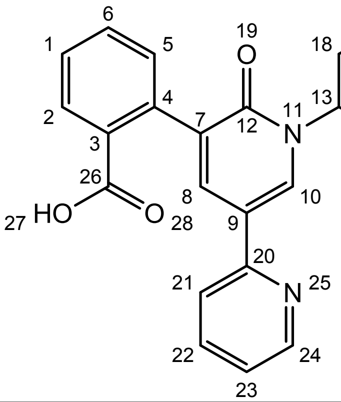

Position	^{1}H (δ ppm)	^{13}C (δ ppm)	COSY	HSQC
1	7.65 – 7.53 (m)	131.37	H-5, H-6	-
2	7.81 (t)	129.65	H-1	C-2
3		134.00	-	-
4	-	137.58	-	-
5	7.65 – 7.53 (m)	132.29	H-1, H-6	C-5
6	7.65 – 7.53 (m)	132.57	H-1, H5	C-6
7	-	128.88	-	-
8	8.20 (d)	135.25	H-10	C-8
9	-	117.79	-	-
10	8.32 (d)	136.65	H-8	C-10
12	-	160.84	-	-
13	-	141.53	-	-
14/18	7.48 – 7.44 (m)	127.25	H-15/H-17, H-16	C-14/C-18
15/17	7.48 – 7.44 (m)	129.65	H-14/H-18, H-16	C-15/C-17
16	7.48 – 7.44 (m)	128.51	H-14/H-18, H-15/H-17	C-16
20	-	153.02	-	
21	7.95 (d)	122.56	H-22	C-21
22	7.81 (t)	137.86	H-21, H-23	C-22
23	7.29 – 7.27 (m)	122.56	H-22, H-24	C-23
24	8.55(d)	149.78	H-23	C-24
26	-	168.95	-	-
27	-	-	-	-

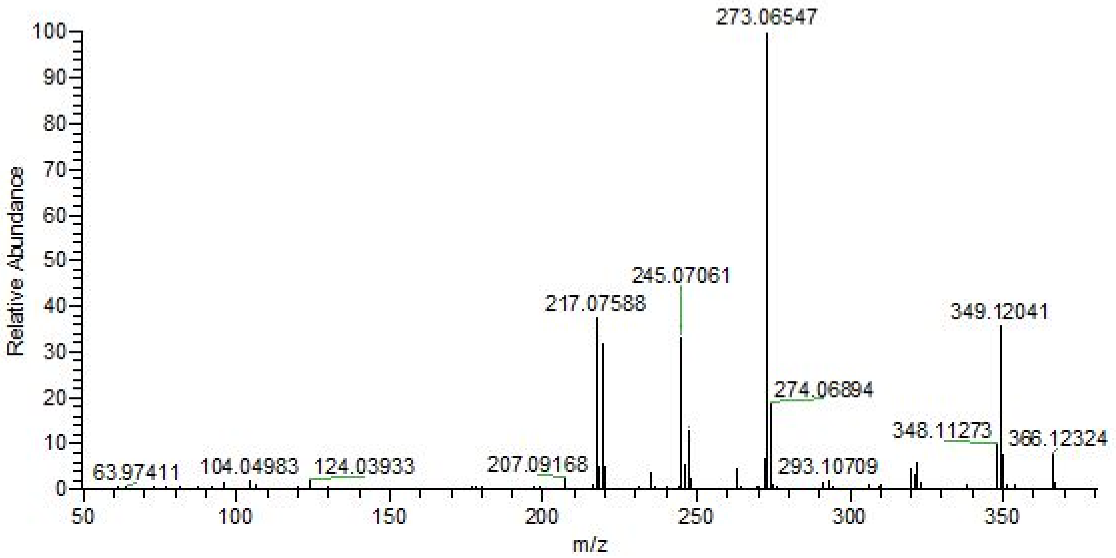

Figure S16. MS/MS spectrum of DP-3.

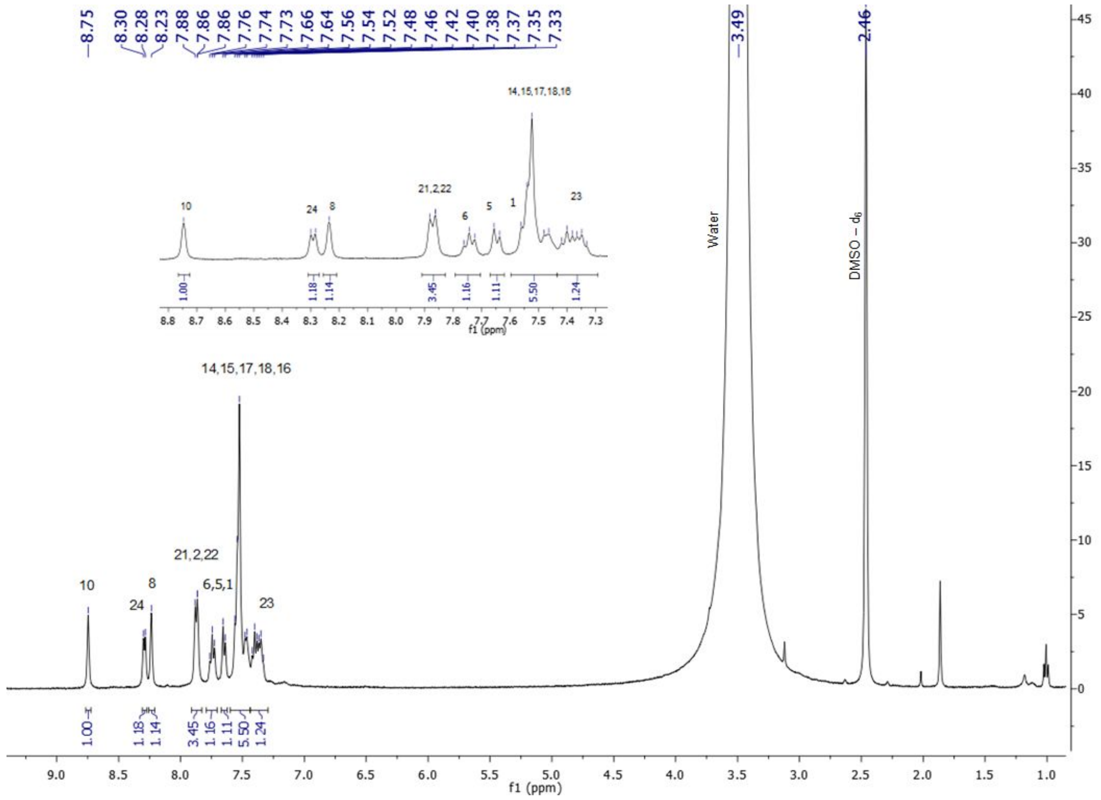

Figure S17. ^{1}H NMR spectrum of DP-3 in DMSO-d_6.

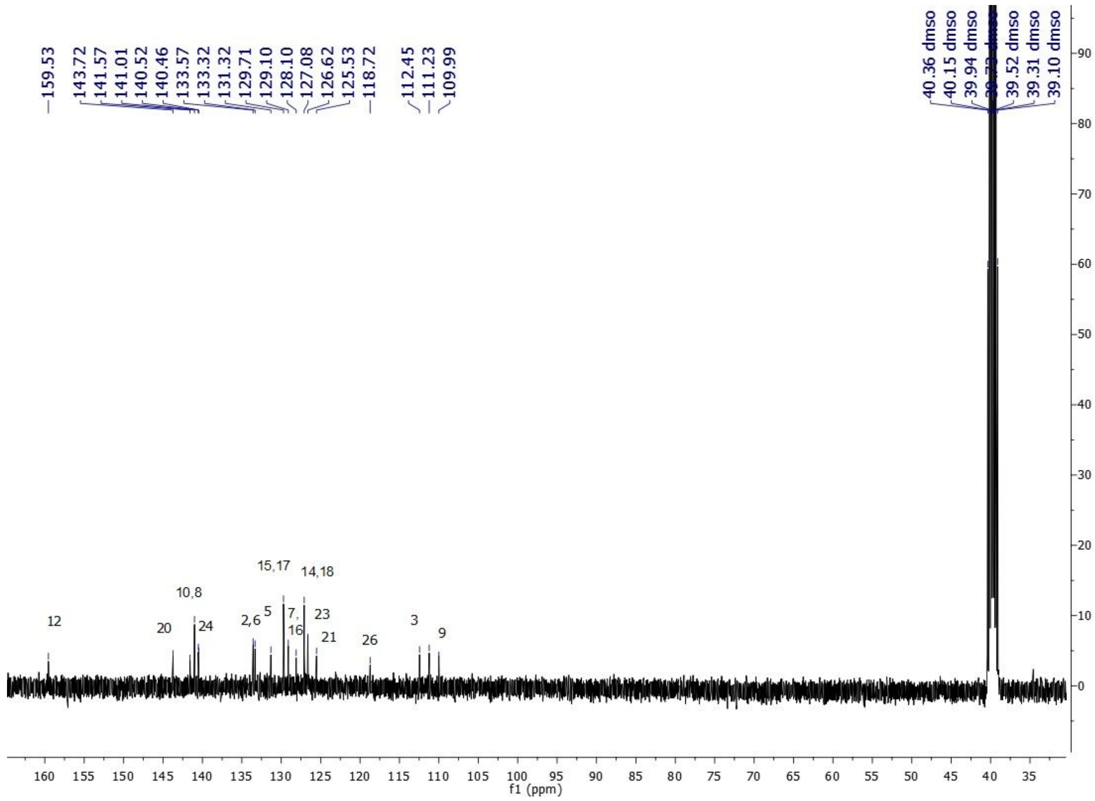

Figure S18. ^{13}C NMR spectrum of DP-3 in DMSO-d_6.

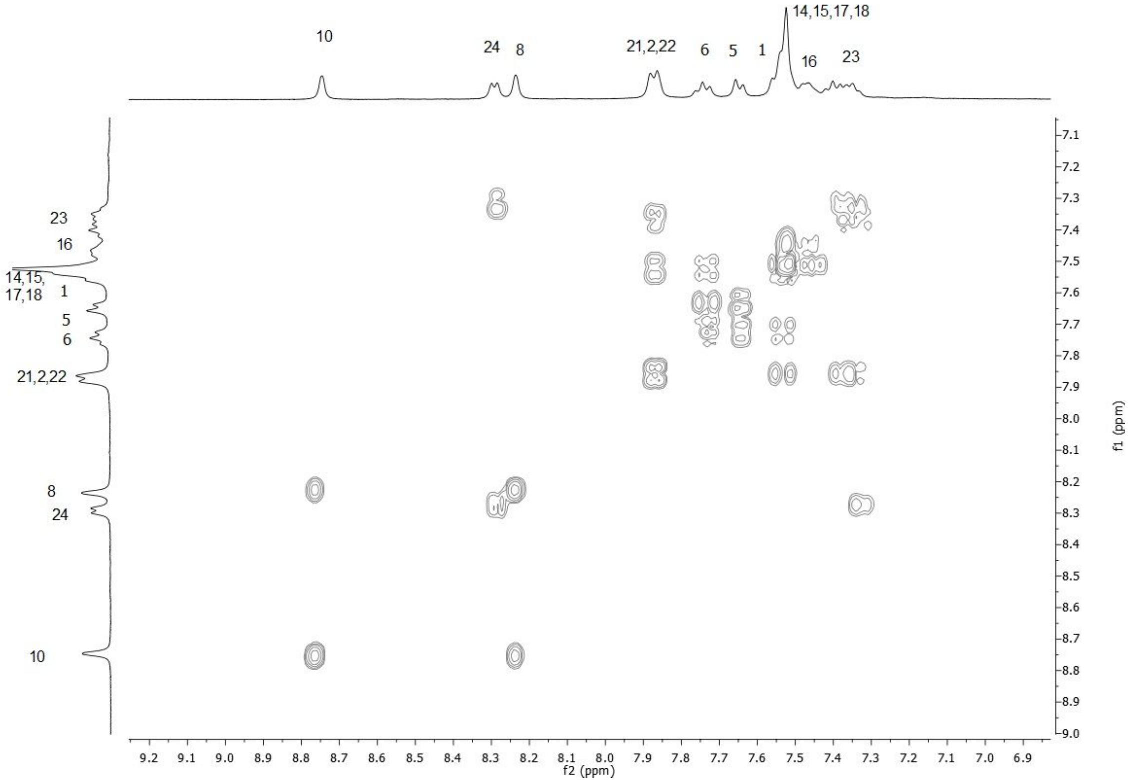

Figure S19. COSY NMR spectrum of DP-3 in DMSO-d_6.

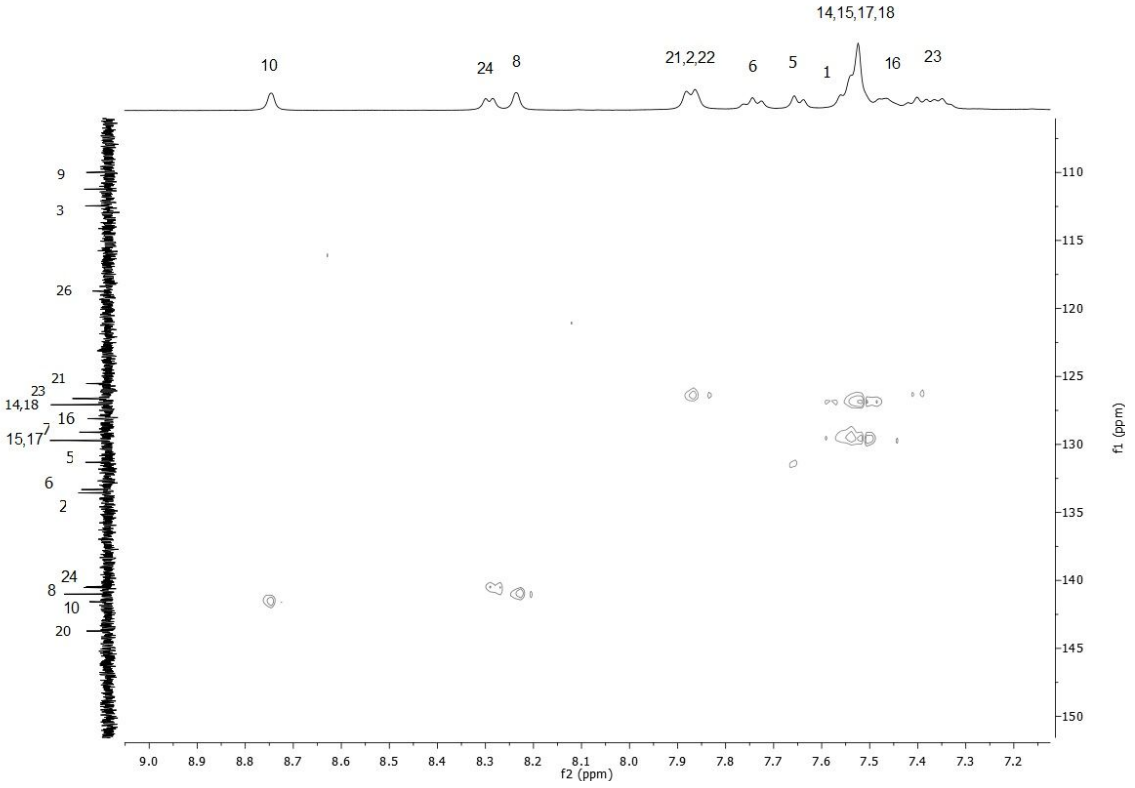

Figure S20. HSQC NMR spectrum of DP-3 in DMSO-d_6.

Table S5. ^{1}H, ^{13}C, COSY and HSQC data of DP-3 in DMSO-d_6.

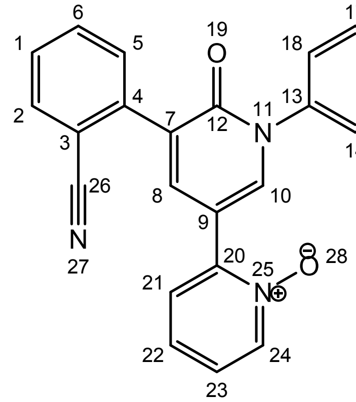

Position	^{1}H (δ ppm)	^{13}C (δ ppm)	COSY	HSQC
1	7.60 – 7.44 (m)		H-2, H-6	
2	7.91 – 7.83 (m)	133.57	H-1	C-2
3	-	112.45	-	C-3
4	-		-	
5	7.65 (d)	131.32	H-6	C-5
6	7.74 (t)	133.32	H-1, H-5	C-6
7	-	129.10	-	C-7
8	8.23 (s)	140.52	H-10	C-8
9	-	110.28	-	C-9
10	8.75 (s)	141.01	H-8	C-10
12	-	159.53	-	C-12
13	-		-	
14/18	7.60 – 7.44 (m)	127.08	H-15/H-17, H-16	C-14/C-18
15/17	7.60 – 7.44 (m)	129.71	H-14/H-18, H-16	C-15/C-18
16	7.60 – 7.44 (m)	128.10	H-14/H-18, H-15/H-17	C-16
20	-	143.72	-	C-20
21	7.91 – 7.83 (m)	125.53	H-22	C-21
22	7.91 – 7.83 (m)		H-21, H-23	
23	7.38 (dt)	126.63	H-22, H-24	C-23
24	8.29 (d)	140.46	H-22, H-23	C-24
26	-	118.72	-	C-26
27	-	-	-	-

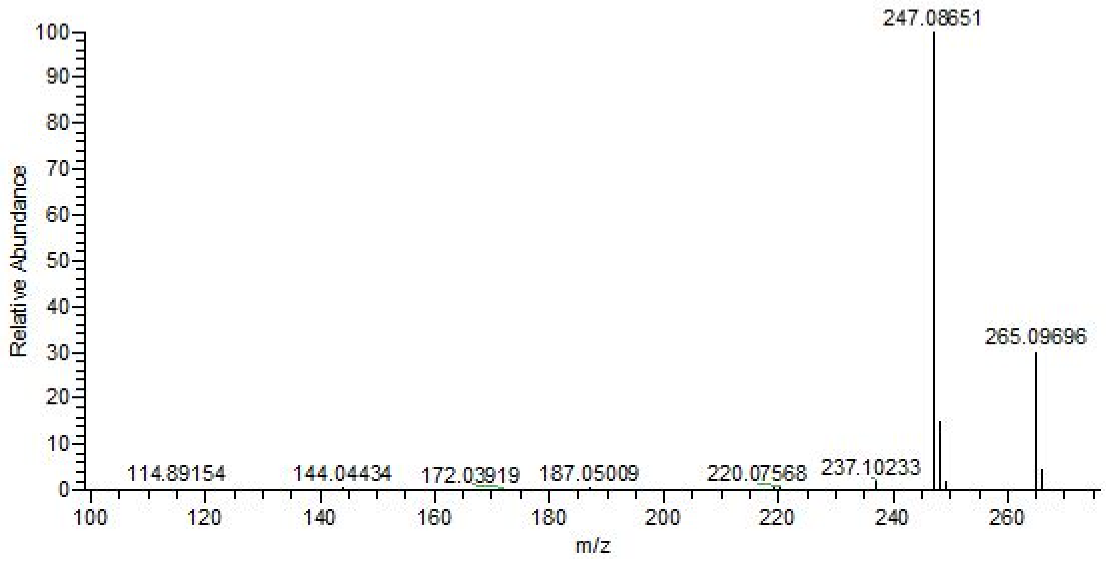

Figure S21. MS/MS spectrum of DP-4.

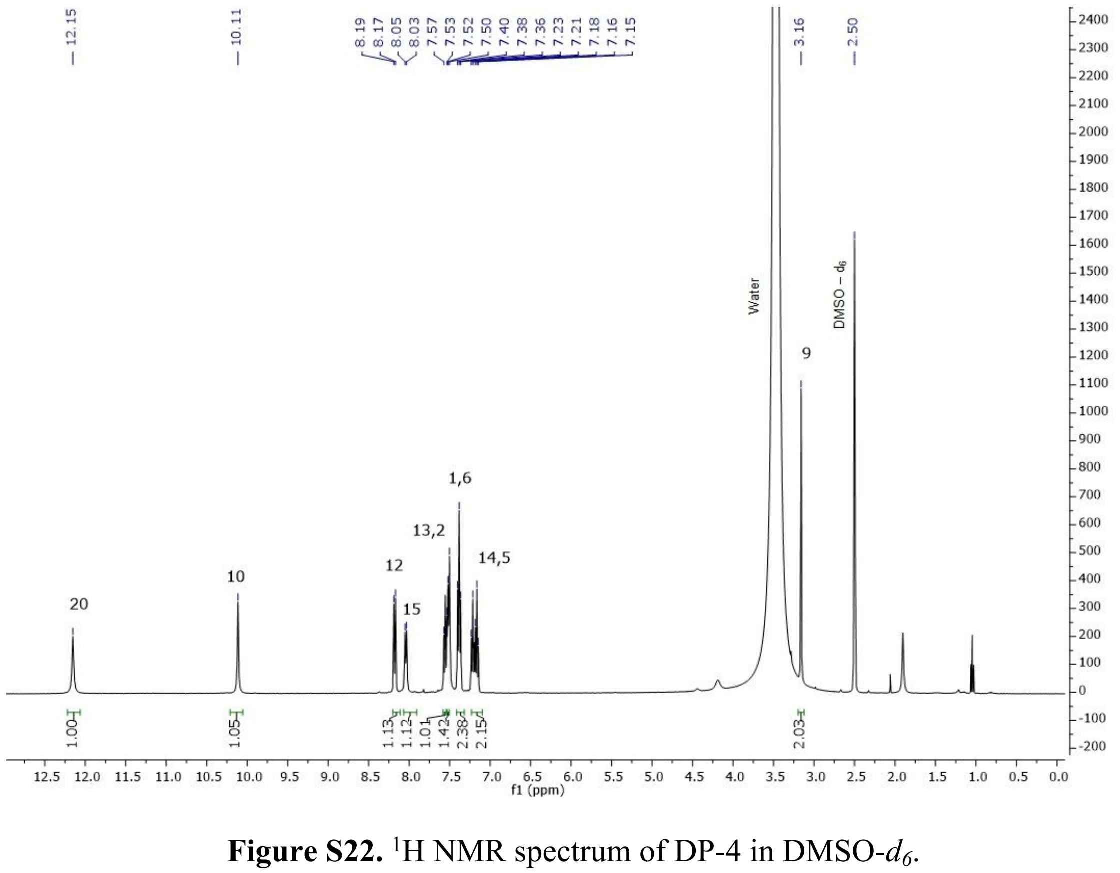

Figure S22. ^{1}H NMR spectrum of DP-4 in DMSO-d_6.

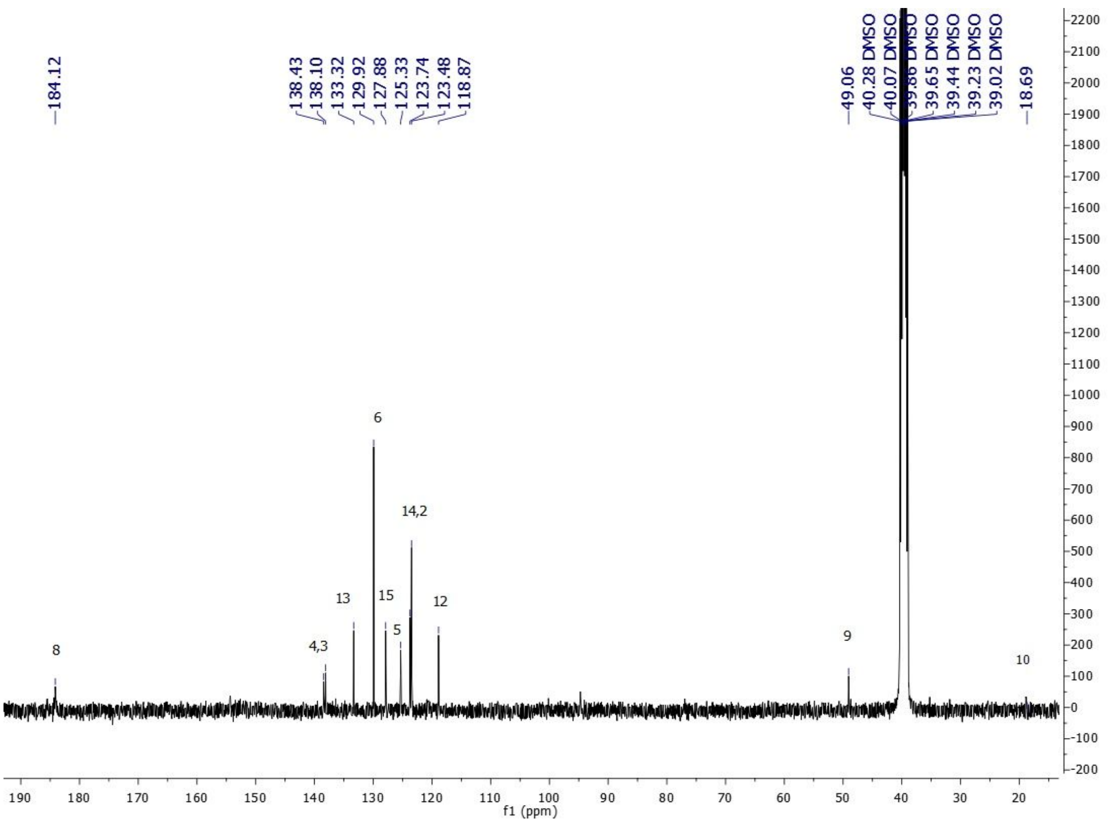

Figure S23. ^{13}C NMR spectrum of DP-4 in DMSO-d_6.

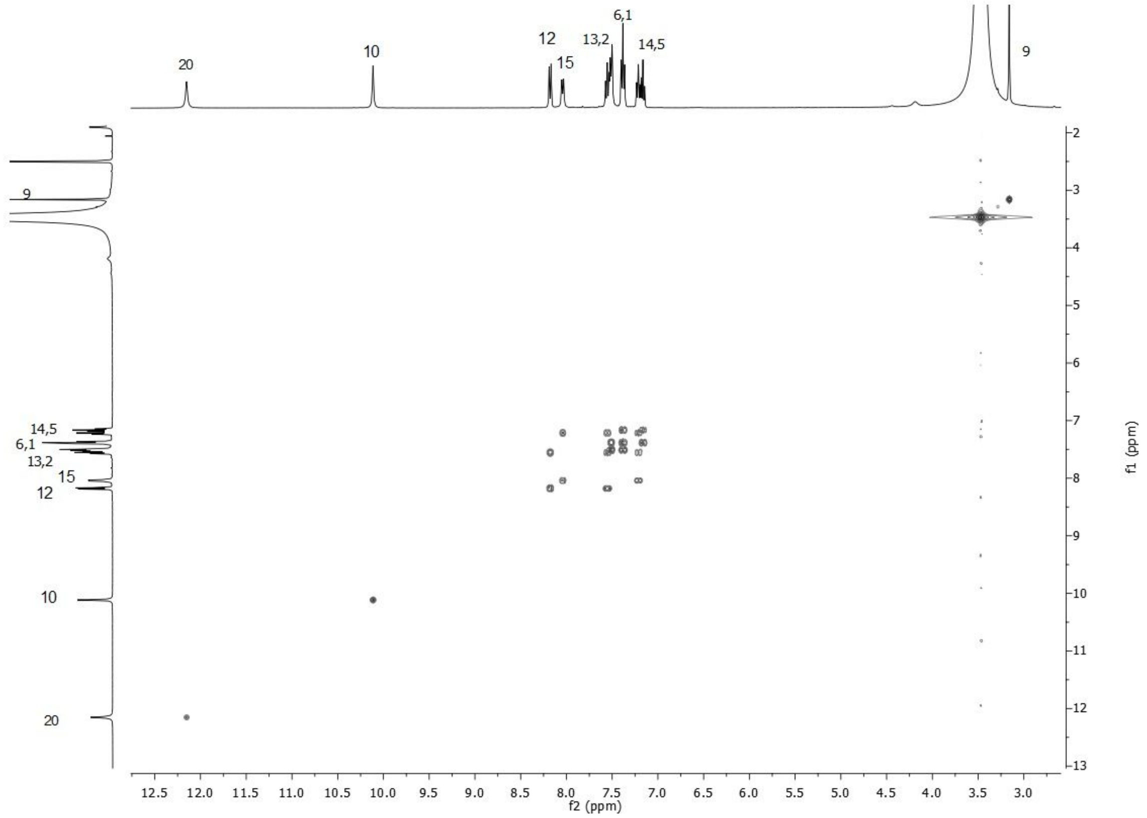

Figure S24. COSY NMR spectrum of DP-4 in DMSO-d_6.

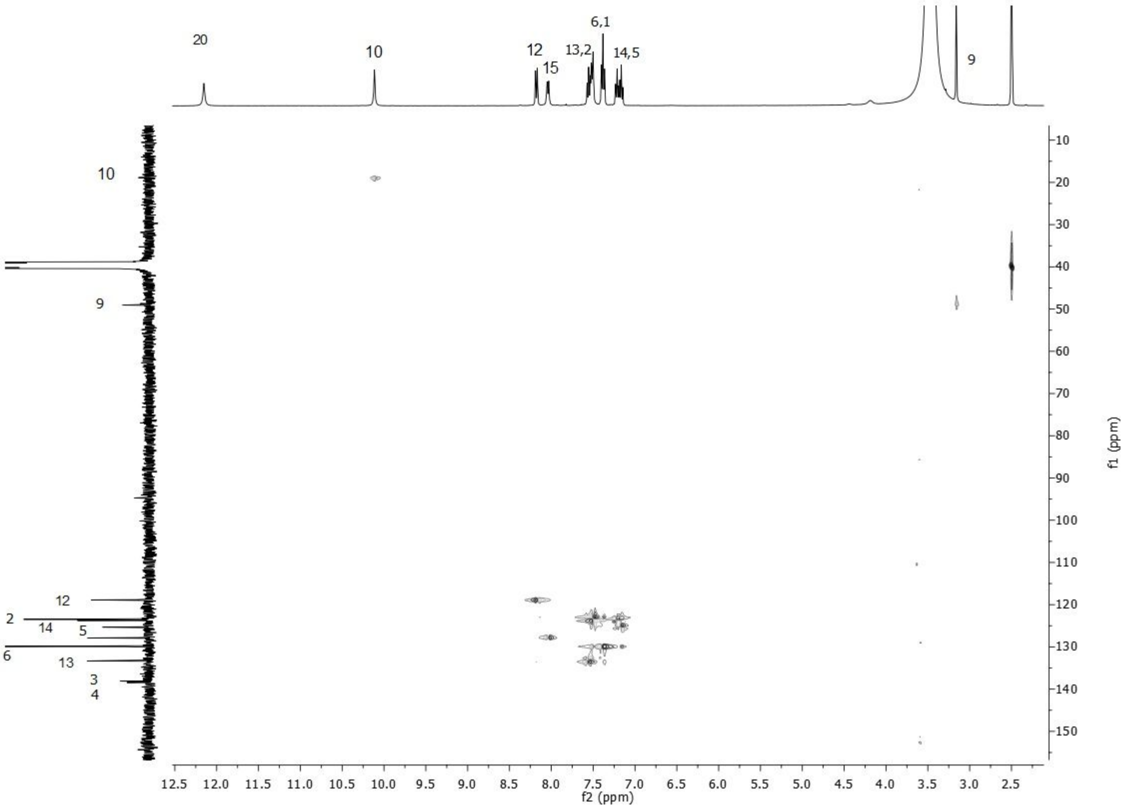

Figure S25. HSQC NMR spectrum of DP4 in DMSO-d_6.

Table S6. ^{1}H, ^{13}C, COSY and HSQC data of DP-4 in DMSO-d_6.

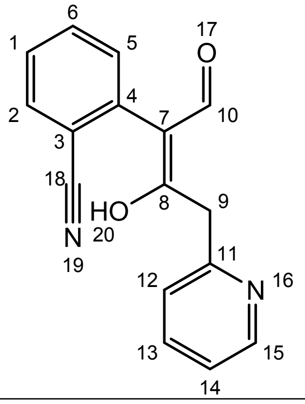

Position	^{1}H (δ ppm)	^{13}C (δ ppm)	COSY	HSQC
1	7.38 (t)	-	H-2, H-6	-
2	7.52 (s)	123.47	H-1	C-2
3	-	138.10	-	-
4	-	138.43	-	-
5	7.23 – 7.10 (m)	125.33	H-6	C-5
6	7.38 (t)	129.92	H-1, H-5	C-6
7	-	-	-	-
8	-	184.12	-	C-8
9	3.16 (s)	49.06	-	C-9
10	10.11 (s)	16.69	-	C-10
11	-		-	-
12	8.20 – 8.11 (m)	118.87	H-13	C-12
13	7.57 (s)	133.32	H-12,H-14	C-13
14	7.23 – 7.10 (m)	123.74	H-15	C-14
15	8.04 (d)	127.88	H-14	C-15
16	-	-	-	-
17	-	-	-	-
18	-	-	-	-
19	-	-	-	-
20	12.15 (s)	-	-	-

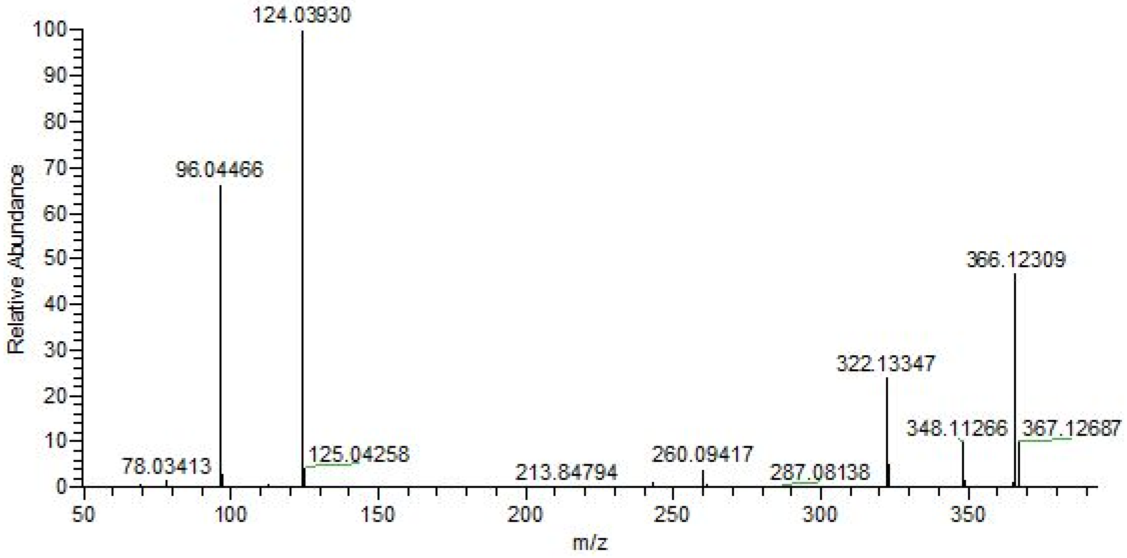

Figure S26. MS/MS spectrum of DP-5.

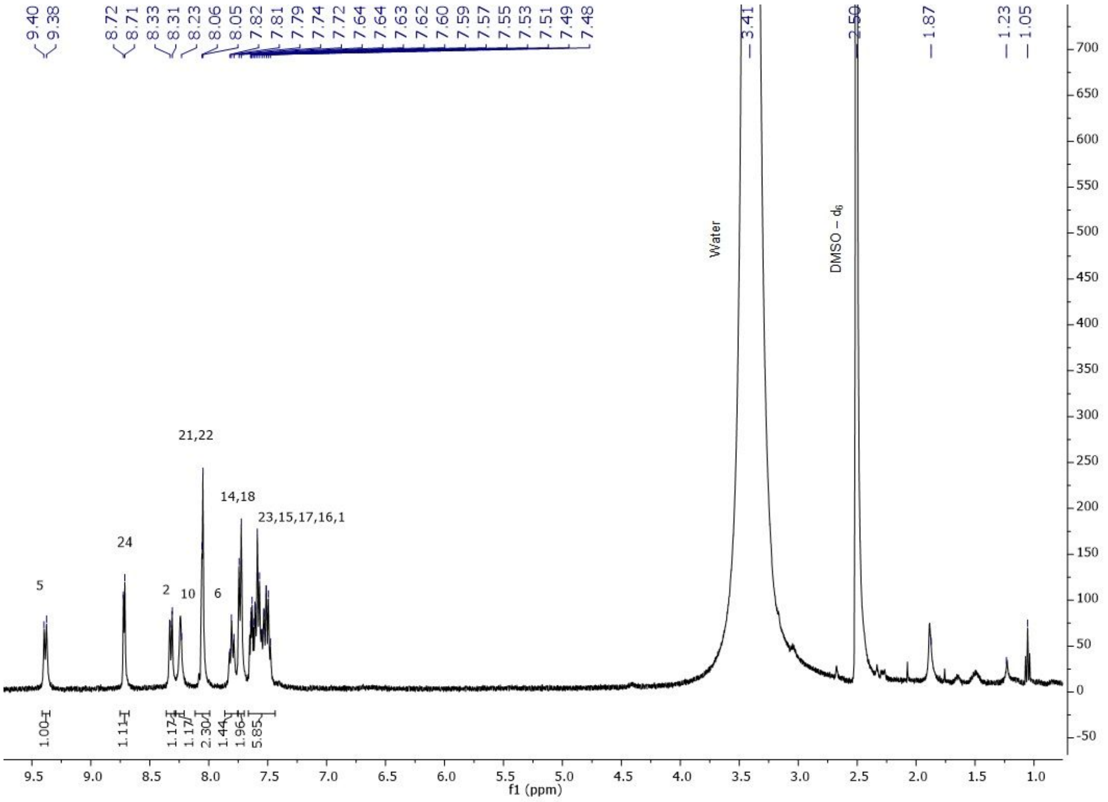

Figure S27. ^{1}H NMR spectrum of DP-5 in DMSO-d_6.

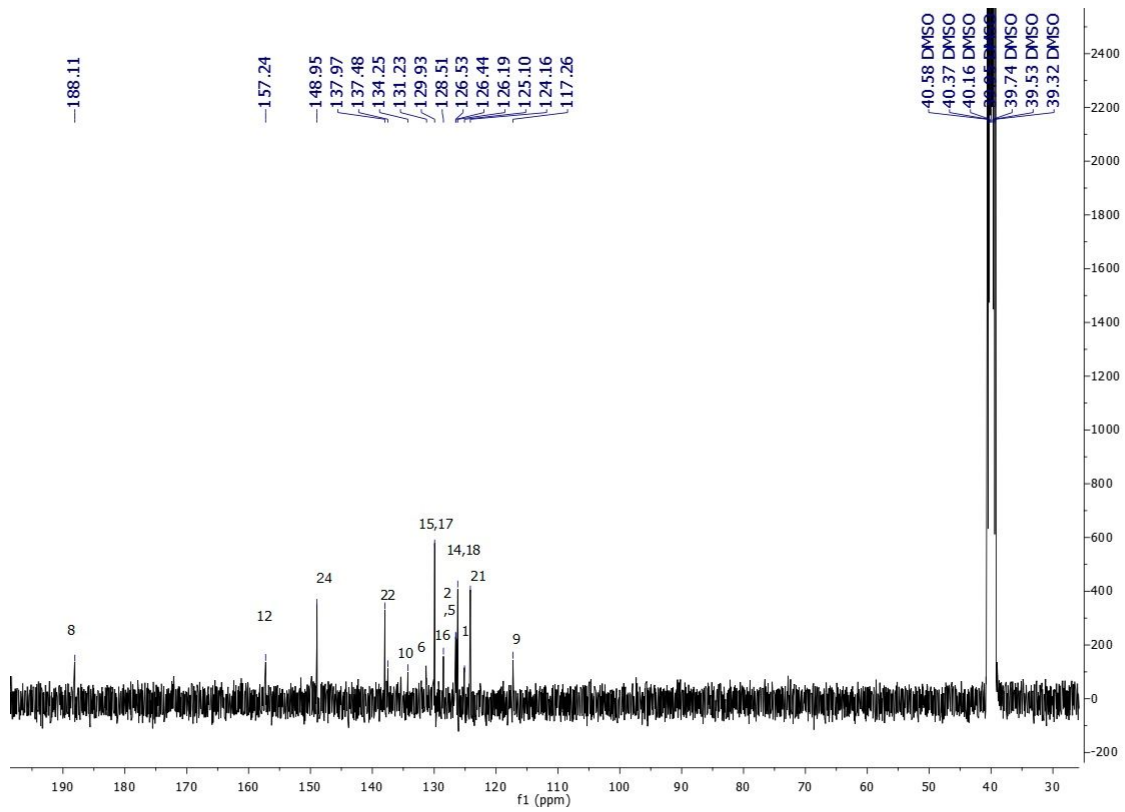

Figure S28. ^{13}C NMR spectrum of DP-5 in DMSO-d_6.

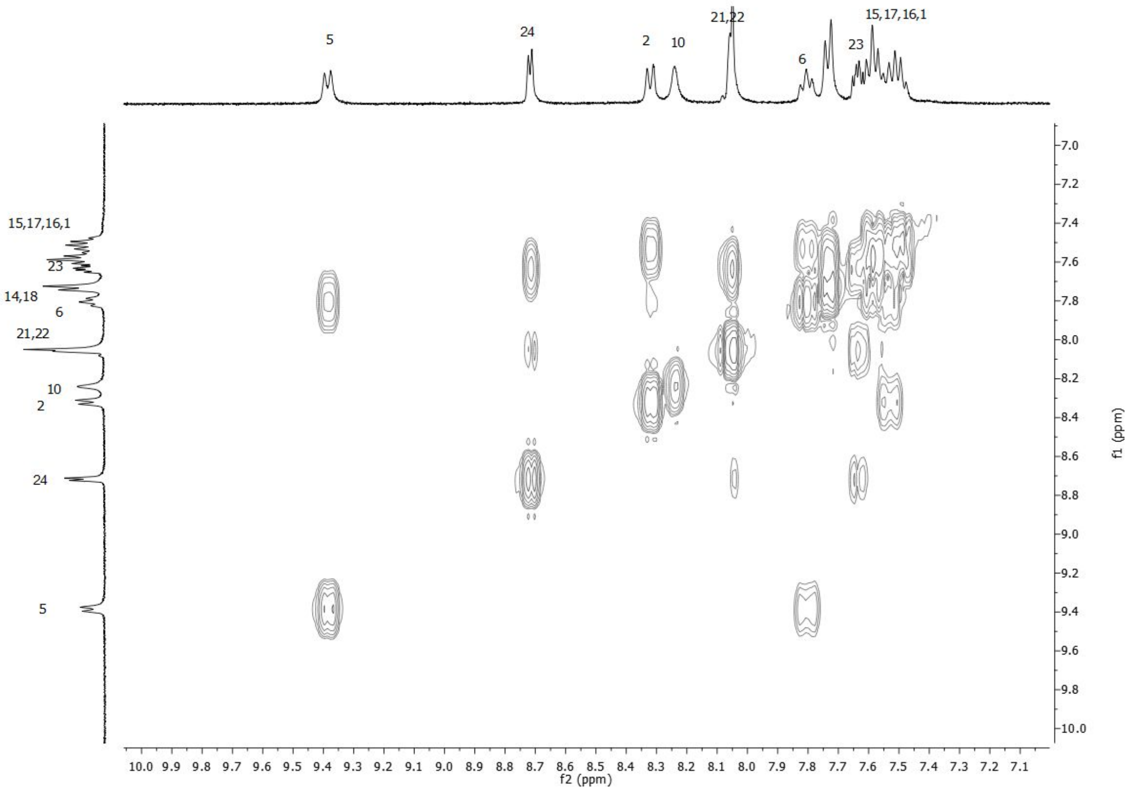

Figure S29. COSY NMR spectrum of DP5 in DMSO-d_6.

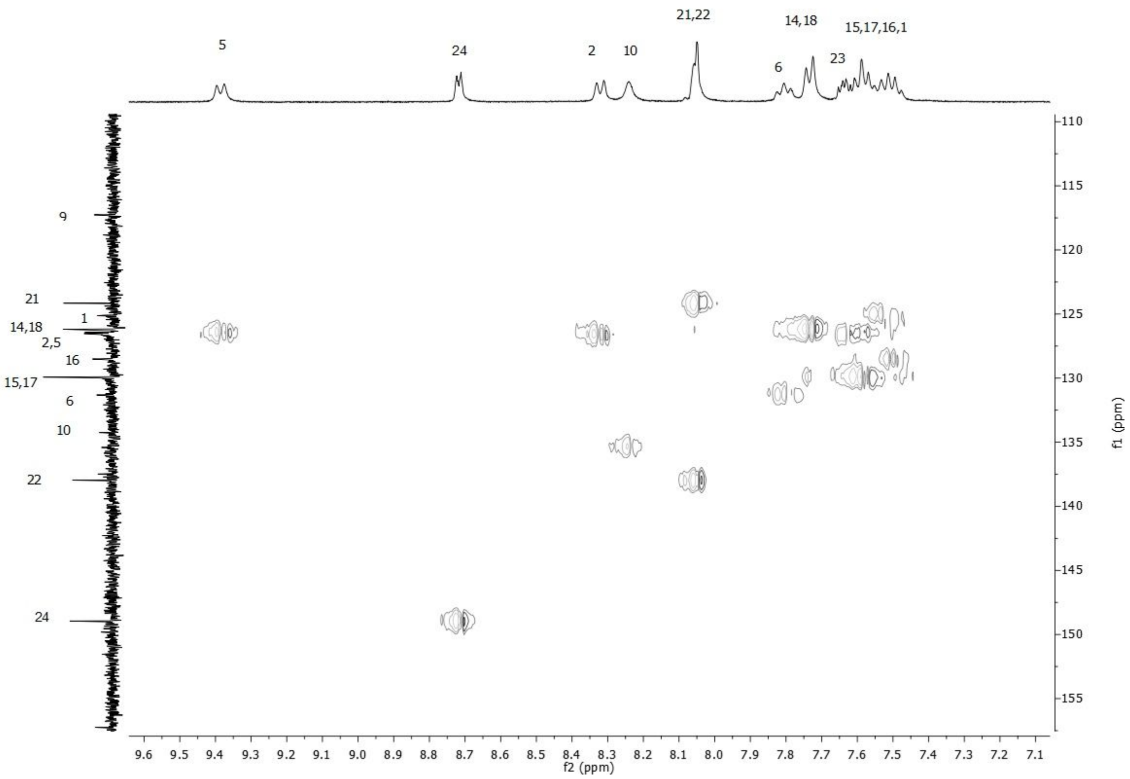

Figure S30. HSQC NMR spectrum of DP5 in DMSO-d_6.

Table S7. ^{1}H, ^{13}C, COSY and HSQC data of DP5 in DMSO-d_6.

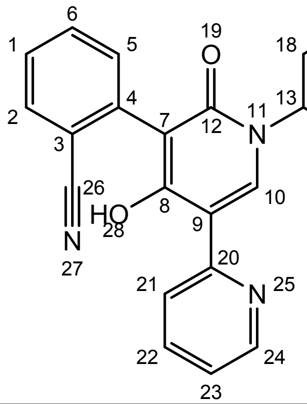

Position	^{1}H (δ ppm)	^{13}C (δ ppm)	COSY	HSQC
1	7.66 – 7.44 (m)	-	H-2	-
2	8.32 (d)	126.53	H-1	C-2
3	-	-	-	-
4	-	-	-	-
5	9.39 (d)	126.44	H-6	C-5
6	7.86 – 7.75 (m)	131.31	H-5	-
7	-	-	-	-
8	-	157.24	-	-
9	-	117.26	-	-
10	8.23 (s)	134.25	-	C-10
12	-	188.11	-	-
13	-	-	-	-
14/18	7.73 (d)	126.19	H-15/H-17, H-16	C-14/C-18
15/17	7.66 – 7.44 (m)	129.93	H-14/H-18, H-16	C-15/C-17
16	7.66 – 7.44 (m)	128.51	H-14/H-18, H-15/H-17	C-16
20	-	-	-	-
21	8.05 (d)	-	H21, H-23, H-24	C-21
22	8.05 (d)	137.97	H21, H-23, H-24	C-22
23	7.66 – 7.44 (m)	-	H21, H-22, H-24	-
24	8.72 (d)	148.95	H21, H-22, H-23	C-24
26	-	-	-	-
27	-	-	-	-
28[a]	-	-	-	-

[a] H-28 (–OH): not observed due to exchange broadening / solvent exchange.

Table S8. *In silico* toxicity evaluation Perampanel and its five degradation products.

Compound	ICH M7 evaluation				Third software mutagenicity evaluation	General Toxicity		
	Mutagenicity Method I	Mutagenicity Method II*	Carcinogenicity	Classification		Cardiotoxicity**	Hepatotoxicity	Acute toxicity (LD50; mg/kg)
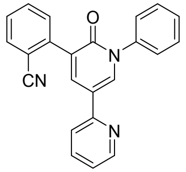 Perampanel (PER)	no	no	no	Class 5	no	no	**yes**	1935
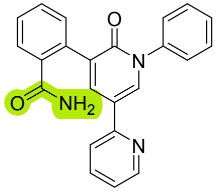 DP-1	no	no	no	Class 5	no	no	**yes**	2480
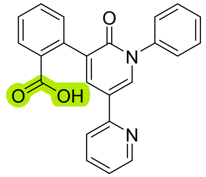 DP-2	no	no	no	Class 4	no[‡]	no	**yes**	1393

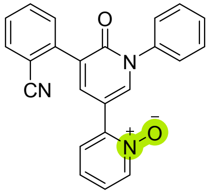<br>DP-3	no	no	no	Class 5	no	no	**yes**	2330
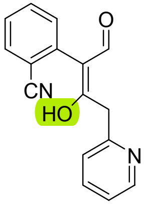<br>DP-4	no	no	no	Class 4	no	no	no	1700
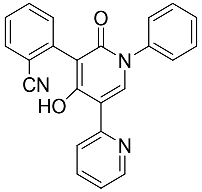<br>DP-5	no	no	no	Class 4	no	no	**yes**	940

* *Low reliability*

** *Cardiotoxicity using statistical and expert-rule methods.*

+ *Reliability: the predicted compound is outside the Applicability Domain of the model.*

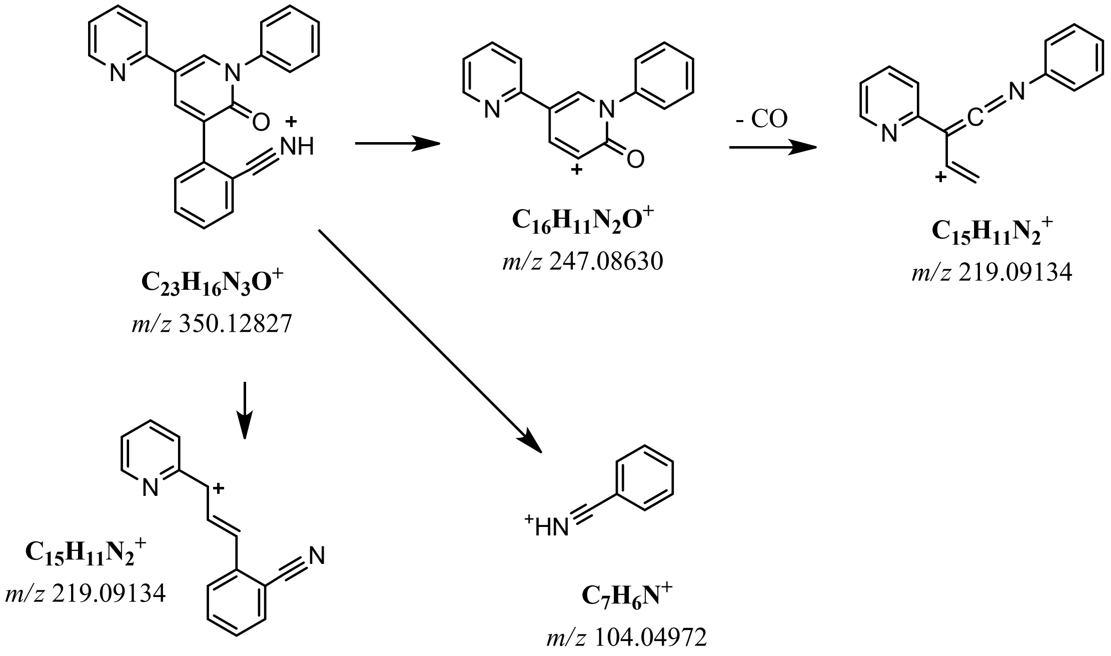

Figure S31. Proposed mass fragmentation pathways of Perampanel.

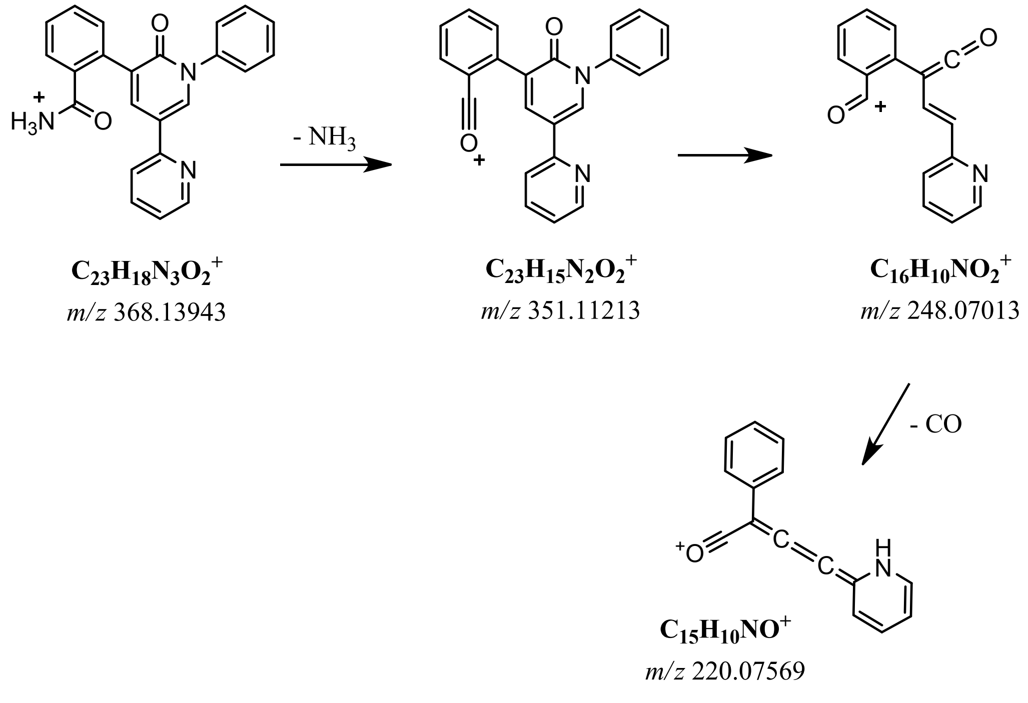

Figure S32. Proposed mass fragmentation pathways of DP-1.

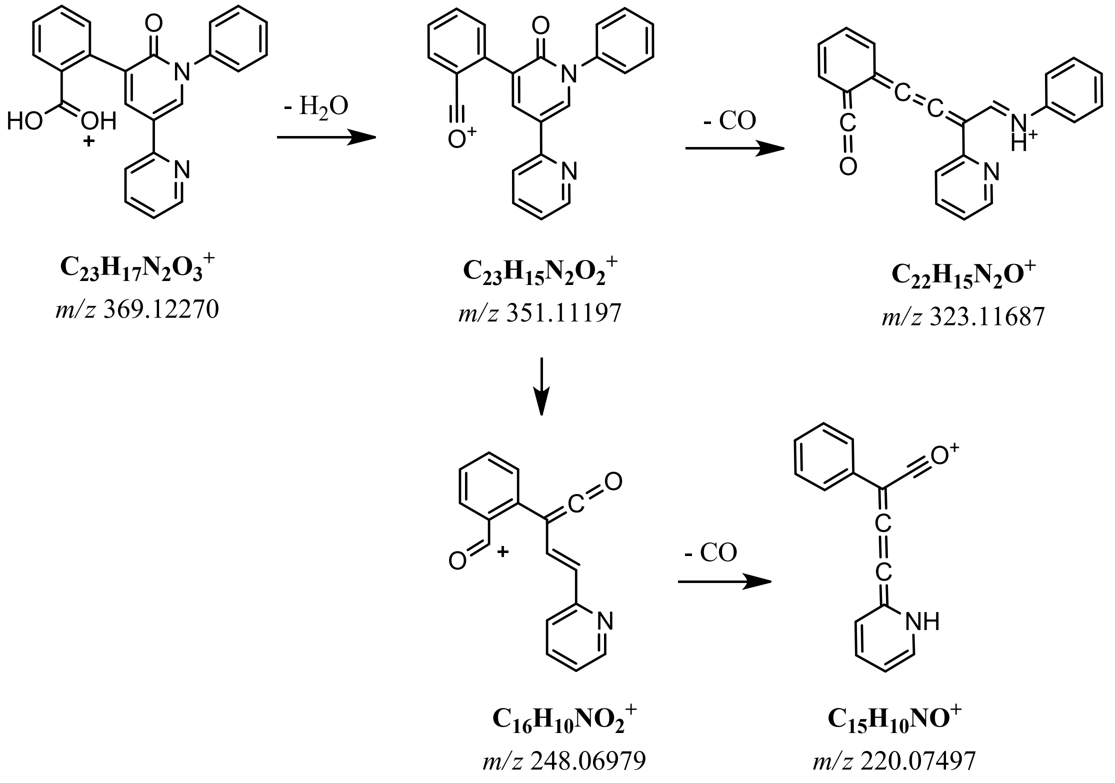

Figure S33. Proposed mass fragmentation pathways of DP-2.

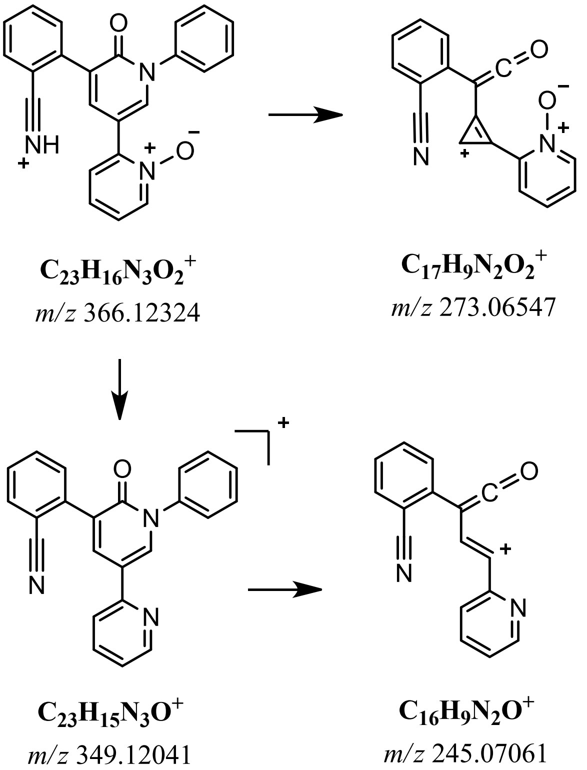

Figure S34. Proposed mass fragmentation pathways of DP-3.

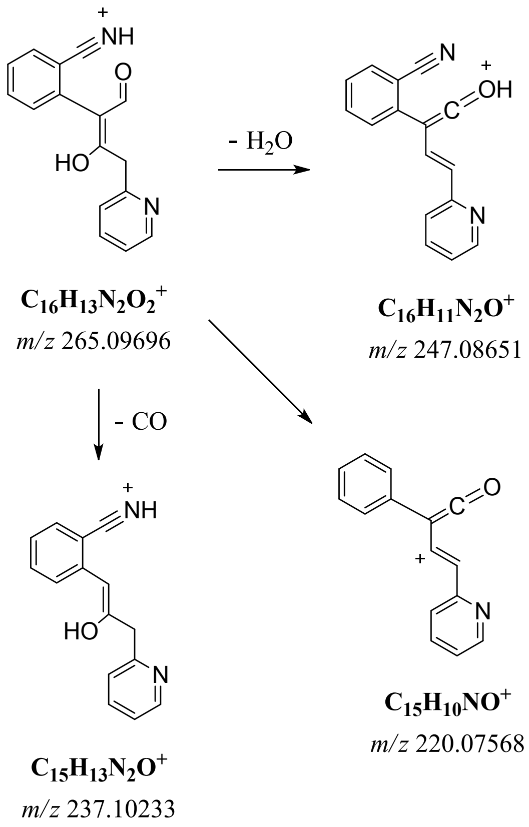

Figure S35. Proposed mass fragmentation pathways of DP-4.

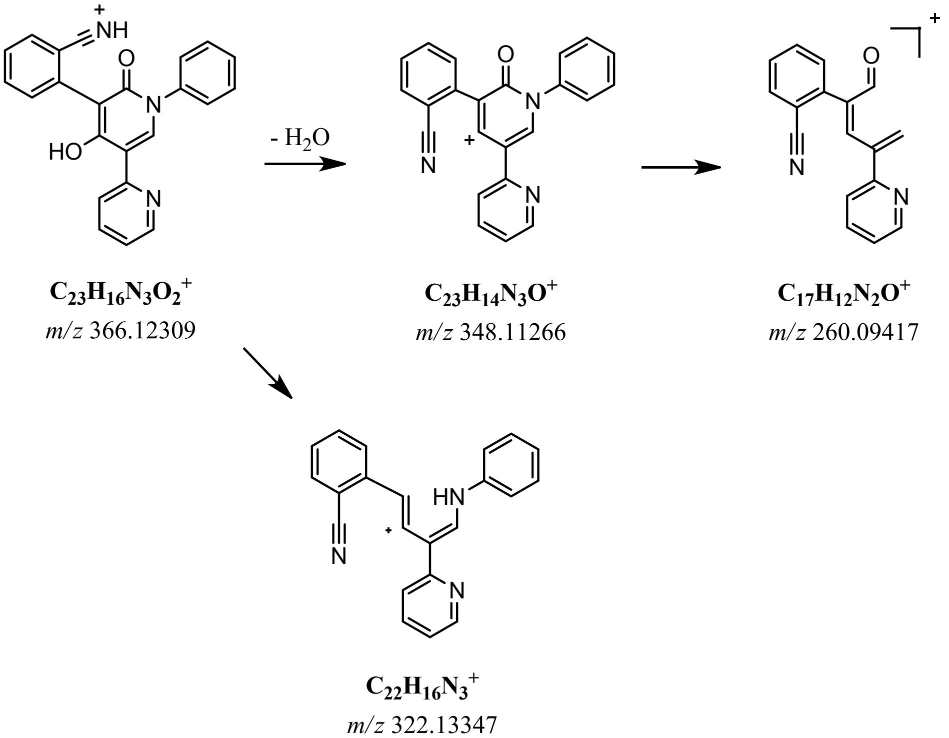

Figure S36. Proposed mass fragmentation pathways of DP-5.

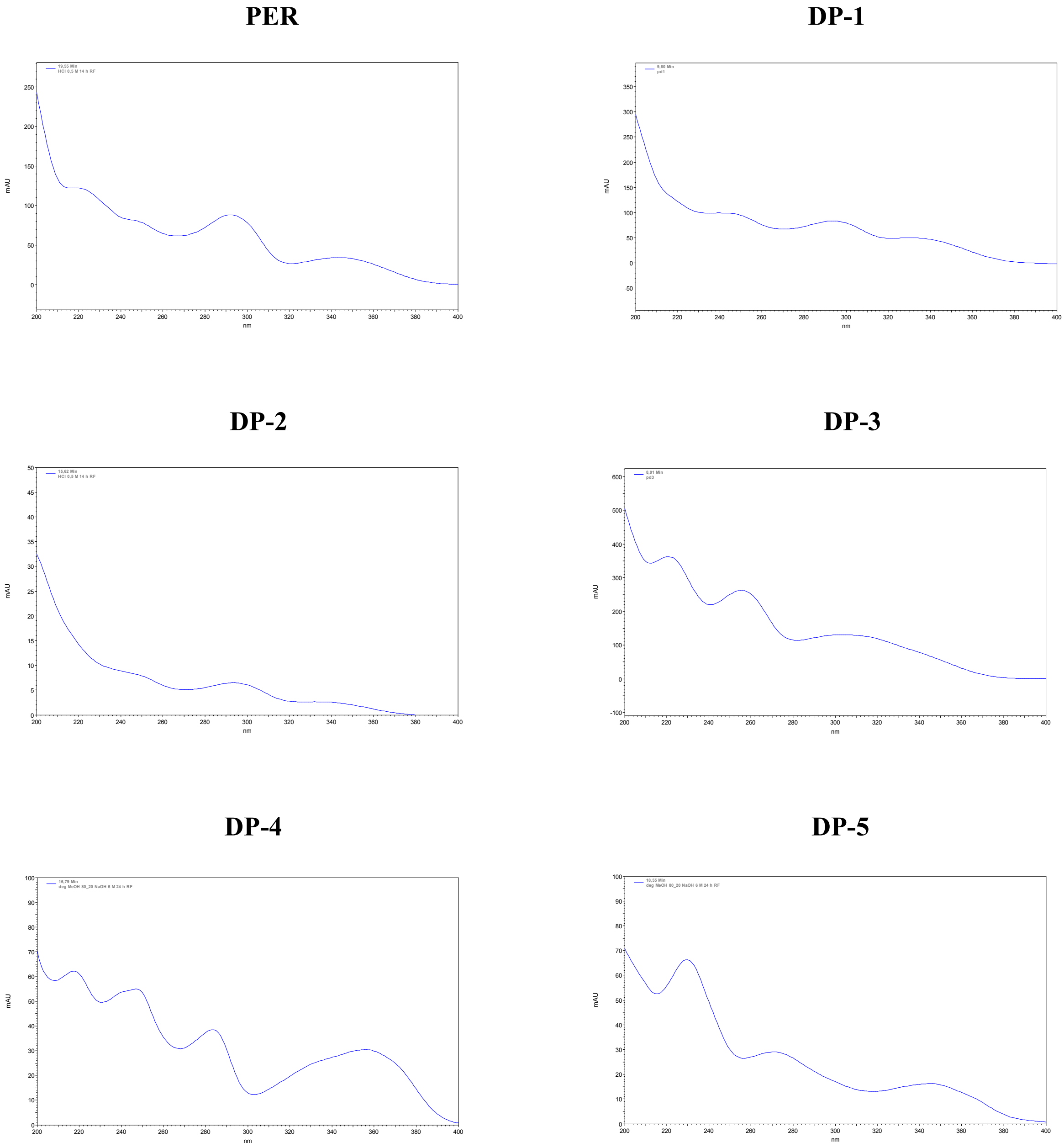

Figure S37. UV absorption spectra at 290 nm of PER and its degradation products (DP-1 to DP-5) recorded using the HPLC–DAD system.
